# Supplementary material for: Cyclopentadienyl coordination induces unexpected ionic Am−N bonding in an americium bipyridyl complex
Source: Nat Commun. 2022 Jan 11;13:201. doi: 10.1038/s41467-021-27821-4 (PMC8752859; doi:10.1038/s41467-021-27821-4)
Supplement: Supplementary file 1 — Supplementary Information [file 41467_2021_27821_MOESM1_ESM.pdf]

Cyclopentadienyl Coordination Induces Unexpected Ionic Am−N Bonding in an Americium Bipyridyl Complex

Brian N. Long,[1] María J. Beltrán-Leiva,[1] Cristian Celis-Barros,[1] Joseph M. Sperling,[1] Todd N. Poe,[1] Ryan E. Baumbach,[2] Cory J. Windorff,[1,3] Thomas E. Albrecht-Schönzart[1*]

[1]Department of Chemistry and Biochemistry, Florida State University, 95 Chieftan Way, Tallahassee, Florida 32306

[2]National High Magnetic Field Laboratory, 1800 E. Paul Dirac Drive, Tallahassee, FL 32310

[3]Department of Chemistry and Biochemistry, New Mexico State University, MSC 3C, PO box 30001, Las Cruces, New Mexico, 88003

*To whom correspondence should be addressed: talbrechtschoenzart@gmail.com

Supplementary Information

Table of Contents

Supplementary Note 1: Experimental and Computational Details 3

Supplementary Note 2: Synthesis and Crystal Pictures 8

Supplementary Note 3: Crystallographic Details 11

Supplementary Note 4: Solid-State UV-vis-NIR Spectra 16

Supplementary Note 5: Solution Phase UV-vis-NIR Spectra 24

Supplementary Note 6: ^{1}H NMR Spectra 30

Supplementary Note 7: Magnetic Susceptibility 33

Supplementary Note 8: SO-pDFT and QTAIM 36

Supplementary References 41

Supplementary Note 1: Experimental and Computational Details

^{1}H NMR

^{1}H NMR spectra were collected on a Bruker Avance III 600 MHz NMR Spectrometer operating at 600.13 MHz and referenced to internal solvent resonances at room temperature. **1−Am** was dissolved in C_6D_6 at 80 °C, filtered, and slowly cooled to room temperature. Two samples of **1−Nd** were dissolved in CD_2Cl_2 and THF-d_8, respectively. **1−Nd** and **1−Am** measurements were collected in a Wilmad-LabGlass 5 mm Thin Wall Precision NMR Sample Tube 7” L, 600 MHz. The **1−Am** NMR tube was lined with a SP Scienceware Teflon tube liner for secondary containment purposes.

Solution Phase UV/vis/NIR Spectroscopy

Solution phase UV/Vis/NIR spectra were collected on a Cary 6000i Series UV/Vis/NIR spectrophotometer at ambient temperature with a 300 nm to 1700 nm scan window. **1−Nd** and **1−Am** were taken up in toluene at 80 °C, filtered, and slowly cooled to room temperature. Spectra were collected in a Starna GL14-C screw cap cell with a path length of 0.4 cm. Spectra of **1−Nd** and **1−Am** were collected under inert conditions and again after 24 hours of air exposure.

Solid State UV/vis/NIR Spectroscopy

Solid state UV/Vis/NIR spectra were collected on a CRAIC Technologies 20/20 PV^{TM} Microspectrophotometer utilizing a 75 W xenon light source. In an argon atmosphere glovebox, crystals were prepared on a glass slide in degassed Parabar 10312 oil, brought out of the glovebox, and quickly transferred to a Linkam LTS420 stage. Prior to collecting, the stage was purged for 5

minutes with N_2. Room temperature collections were taken from 320 nm to 1700 nm before cooling the sample to −180 °C at 5 °C/min. Measurements were collected again at −180 °C. Room temperature measurements were collected once more after 24 hours of air exposure to the crystals.

Crystallography

Single crystal X−ray diffraction was obtained on a Bruker D8 Quest X−ray Diffractometer with a IμS X-ray source (Mo K_α; $\lambda = 0.71073$ Å). In an argon atmosphere glovebox, single crystals of **1−Nd** and **1−Am** were gathered under Parabar 10312 oil and placed into a petri dish. The petri dish was taped closed in order to maintain an inert atmosphere as long as possible, and removed from the glovebox. **1−Nd** was collected at 120 K and **1−Am** was collected at 100 K under a N_2 stream. Both crystals were mounted on 75 μm MiTeGen loops. Crystal alignment, unit cell determination, and data integration were performed with APEXIII software. Structural refinement was completed with SHELXTL suite through the OLEX2 GUI.[1,2] Full crystallographic details are available in Supplementary Table 2 and Supplementary Table 3. Cifs are available at CCDC Deposition #'s 2081956 **(1−Nd)** and 2081957 **(1−Am)**.

Magnetic Susceptibility

An empty gel cap was inserted into a straw to serve as a background. Crystals of **1−Nd** (8.3 mg) were sealed in a gel cap, inserted into a straw, and placed into a Quantum Design VSM Physical Property Measurement System. Measurements were completed using an applied magnetic field, $H = 5$ kOe, at temperatures ranging from $T = 1.8 – 300$ K. Evans Method magnetic susceptibility measurements were completed at 293.4 K on a Bruker Avance III 600 MHz NMR Spectrometer operating at 600.13 MHz. Samples of Cp'_3Nd and **1−Nd** in CD_2Cl_2 were placed in

SP Scienceware Teflon tube liners and inserted into Wilmad-LabGlass 5 mm Thin Wall Precision NMR Sample Tubes 7” L, 600 MHz. The outer lining was filled in a 1:1 mixture of CD_2Cl_2 and DCM (Supplementary Figures 23 and 24).

Computational Details

The electronic structures of **1-Nd** and **1-Am** were analyzed through the complete active space self-consistent field (CASSCF) method using the OpenMolcas program.[3,4] The $(Cp'_3U)_2(\mu{-}4,4'{-}bpy)$ (**1−U***) complex,[5] synthesized by Mehdoui in 2013, was also calculated in order to perform a more complete comparison through the available actinide systems.[5] To keep the structure imposed by the crystal packing, no geometry optimizations were performed. Due to the size of the systems, two models were considered to get insight in the effect of the bridge on the electronic structure (Supplementary Figure 26). The first model (M1) consisted of one Cp'_3M (M = Nd^{3+}, Am^{3+}) unit coordinated to pyridine while the second model (M2) consisted of the same unit instead coordinated to 4,4′−bpy.

The initial wavefunctions were obtained at Hartree–Fock (HF) level of theory along with all-electron ANO-RCC Gaussian-type basis sets contracted to TZP quality.[6,7] Scalar relativistic effects were incorporated *via* the second-order Douglas-Kroll-Hess (DKH2) Hamiltonian.[8] The resulting wavefunction was employed to perform state-average (SA) CASSCF calculations. The active space consisted of n electrons in 8 orbitals (n = 5 for **1-Nd, 1−U***, and 8 for **1-Am**)[5] including the *f* shell, plus one bonding ligand orbital with contributions of the $f_{y(y^2-3x^2)}$ orbital (Supplementary Figure 27). The number of configuration state functions (CSFs) and multiplicities calculated in each system are shown in Supplementary Table 1. Attempts to expand the active space in these systems by including the md_{z^2}, md_{xz} and md_{yz} orbitals (m = 5 for **1−Nd** and 6 for

1−**U*** and **1−Am**) with their bonding counterparts, CAS(n,14) (n = 11 for **1−Nd**, **1−U*** and 14 for **1−Am**) were not successful because an imbalance of the active space was produced.[5] Due to previous reports attributing the contraction of some Am–N bonds to backdonation,[9,10] the active spaces **1−Am** were expanded including orbitals from the pyridine and 4,4′−bpy. However, as in the previous case, no reliable results were obtained due to an imbalanced wavefunction. Dynamic correlation was included through multiconfigurational pair-density functional theory (MC-pDFT) using the tPBE on-top functional.[11] The Spin-orbit (SO) coupling was included by state interactions between the CASSCF wavefunctions using the restricted active space state interaction (RASSI) method.[12]

To investigate the origin of the metal (M)–ligand (L) interaction of **1−U***, **1−Am** and **1−Nd**, we harnessed the virtues of the quantum theory of atoms in molecules (QTAIM).[5,13] Within the QTAIM framework, bonds are analyzed based on metrics calculated at the position where forces balance out at a minimum of electron density along the bond path. The metrics typically used to characterize a bond are the concentration of electron density, ρ(r), at the bond critical point (BCP), and the metrics derived from it such as, total energy density, H(r), and ellipticity, ε(r). Localization, λ(r), and delocalization indices, δ(r), are also useful to support the origin of the interactions occurring upon metal coordination. Changes in ρ(r) and δ(r), could, in theory, be related to orbital overlap, if increases in both ρ(r) and δ(r) are observed. Conversely, increases of only δ(r) indicate a better energy match between the metal and ligand orbitals. Energy densities such as potential, V; kinetic, G; and H(r) help to directly address covalency, where a negative value indicates a stabilization of the electron density at the BCP due to an excess of potential energy. Analogously, a positive H(r) term indicates a purely ionic bond, due to an excess of kinetic energy. All QTAIM

metrics were obtained from the scalar-relativistic CASSCF ground-state densities of Mod1 using the AIMAll (version 19.10.12).[14]

Supplementary Table 1. Number of CSF per multiplicity employed in the CASSCF calculations.

**Multiplicity**	**Number of CSFs**		
	**1−Am**	**1−Nd**	**1−U***
Doublets	-	150	150
Triplets	150	-	-
Quartets	-	350	350
Quintets	350	-	-
Septets	63	-	-

Supplementary Note 2: Synthesis and Crystal Pictures

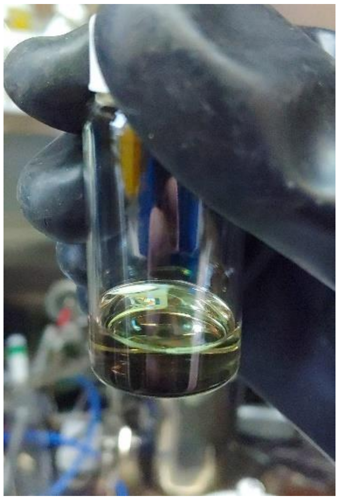

A

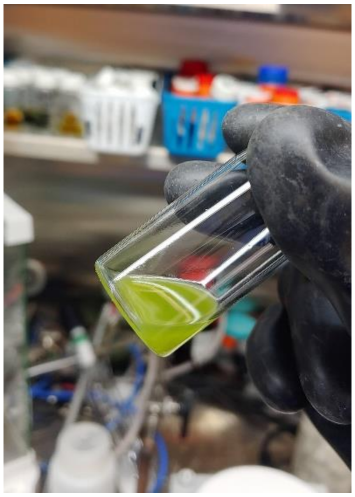

B

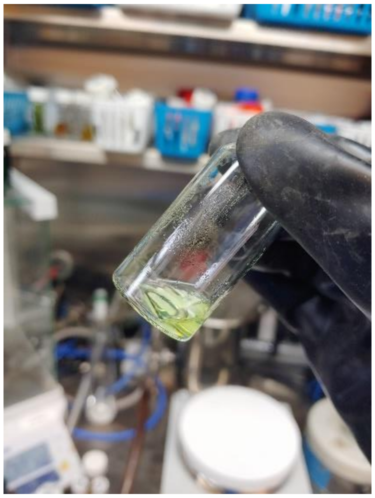

C

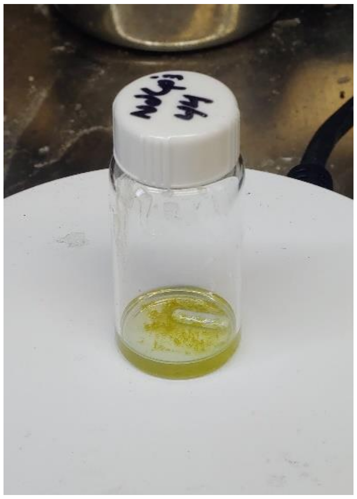

D

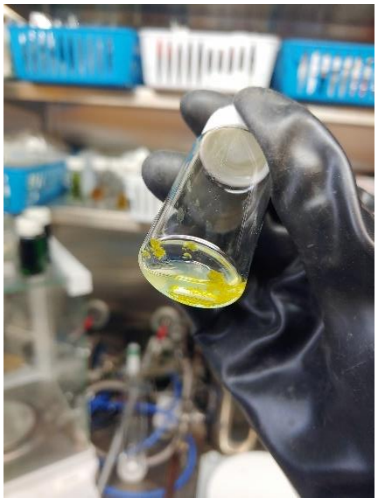

E

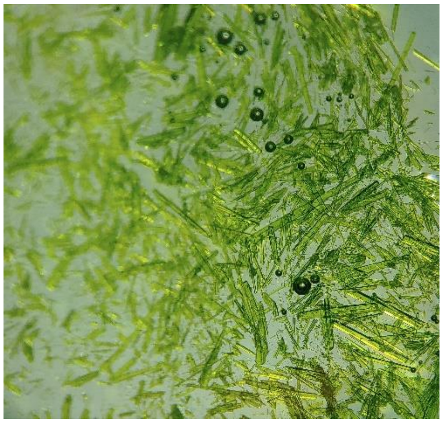

F

Supplementary Figure 1. A. Solution of Cp′$_3$Nd in toluene. **B.** Slurry of (Cp′$_3$Nd)$_2$(μ−4,4′−bpy), **1−Nd**. **C.** Solution of (Cp′$_3$Nd)$_2$(μ−4,4′−bpy), **1−Nd**, in hot toluene. **D.** Crystals of **1−Nd** grown upon cooling to room temperature. **E.** Crystals of **1−Nd** in toluene at room temperature. **F.** Batch of crystals of **1−Nd** used for single-crystal X-ray diffraction and solid-state UV-vis-NIR spectroscopy.

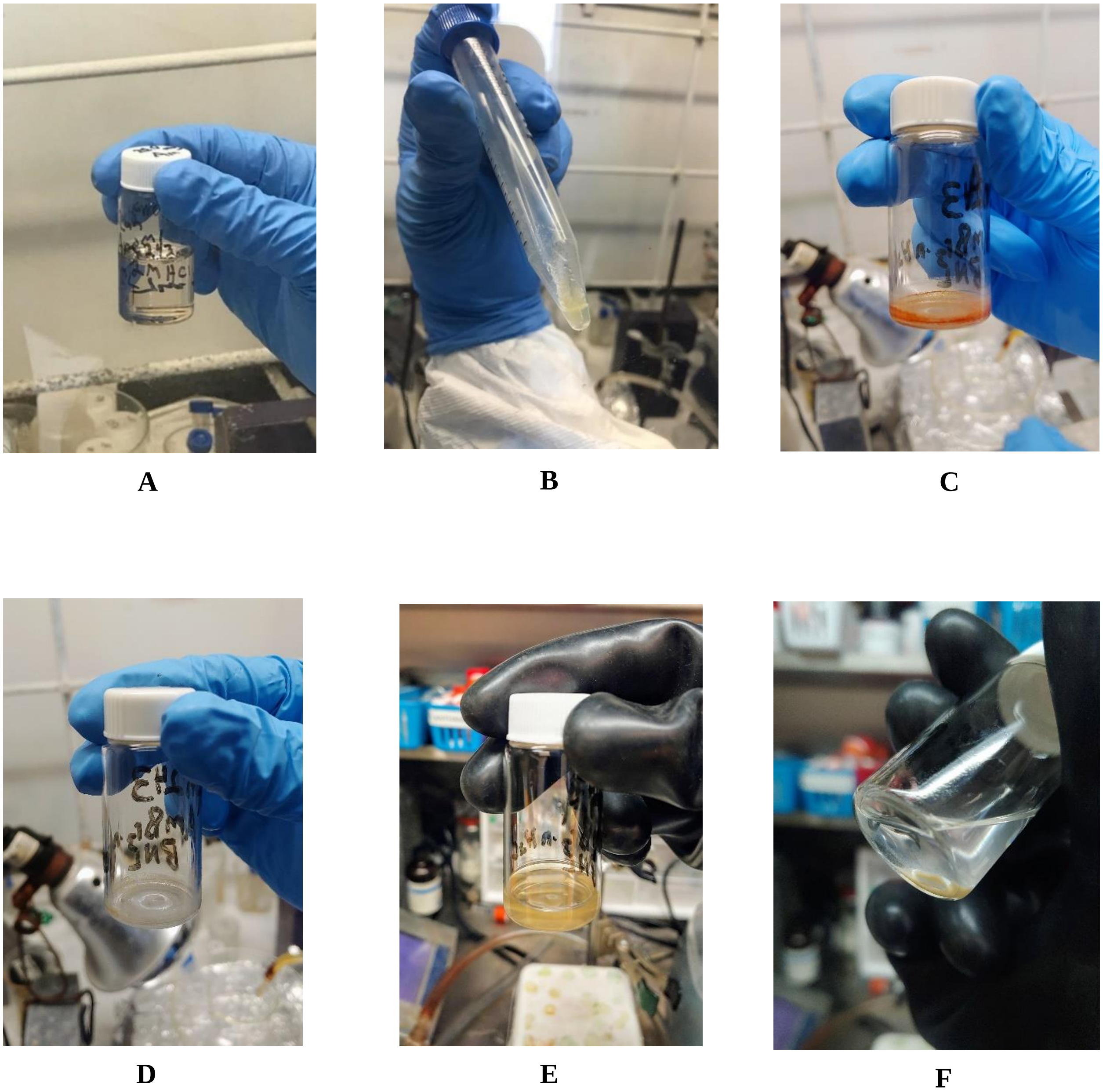

Supplementary Figure 2. A. Stock solution of Am^{3+} (2 mg/mL). **B.** Pellet of $Am(OH)_3$ after centrifuging. **C.** $AmBr_3(H_2O)_n$ before OEt_2 wash. **D.** $AmBr_3(H_2O)_n$ after OEt_2 wash. **E.** $AmBr_3(DME)_n$ in DME/TMS−Br. **F.** $AmBr_3(DME)_n$ in OEt_2/Hexane rinse.

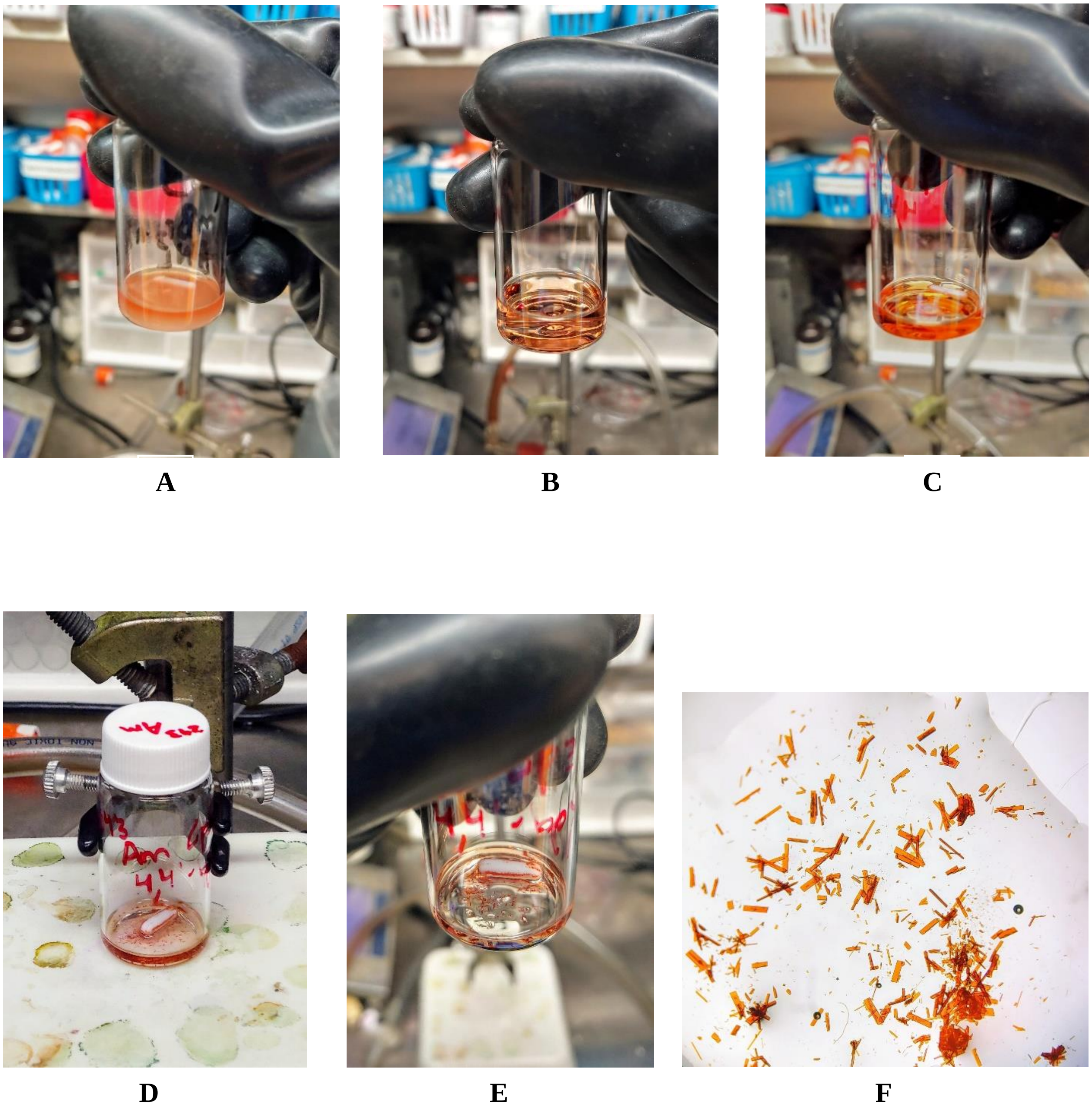

Supplementary Figure 3. A. Addition of KCp′ to $AmBr_3(DME)_n$ resulting in a putative Cp'_3Am. **B.** Filtered solution of Cp'_3Am in hexane. **C.** Solution of $(Cp'_3Am)_2(\mu{-}4,4'{-}bpy)$, **1−Am**, in hot toluene. **D.** Crystals of **1−Am** grown from toluene overnight. **E.** Another batch of crystals of **1−Am**. **F.** Crystals of **1−Am** used for single-crystal X-ray diffraction and solid-state UV-vis-NIR spectroscopy.

Supplementary Note 3: Crystallographic Details

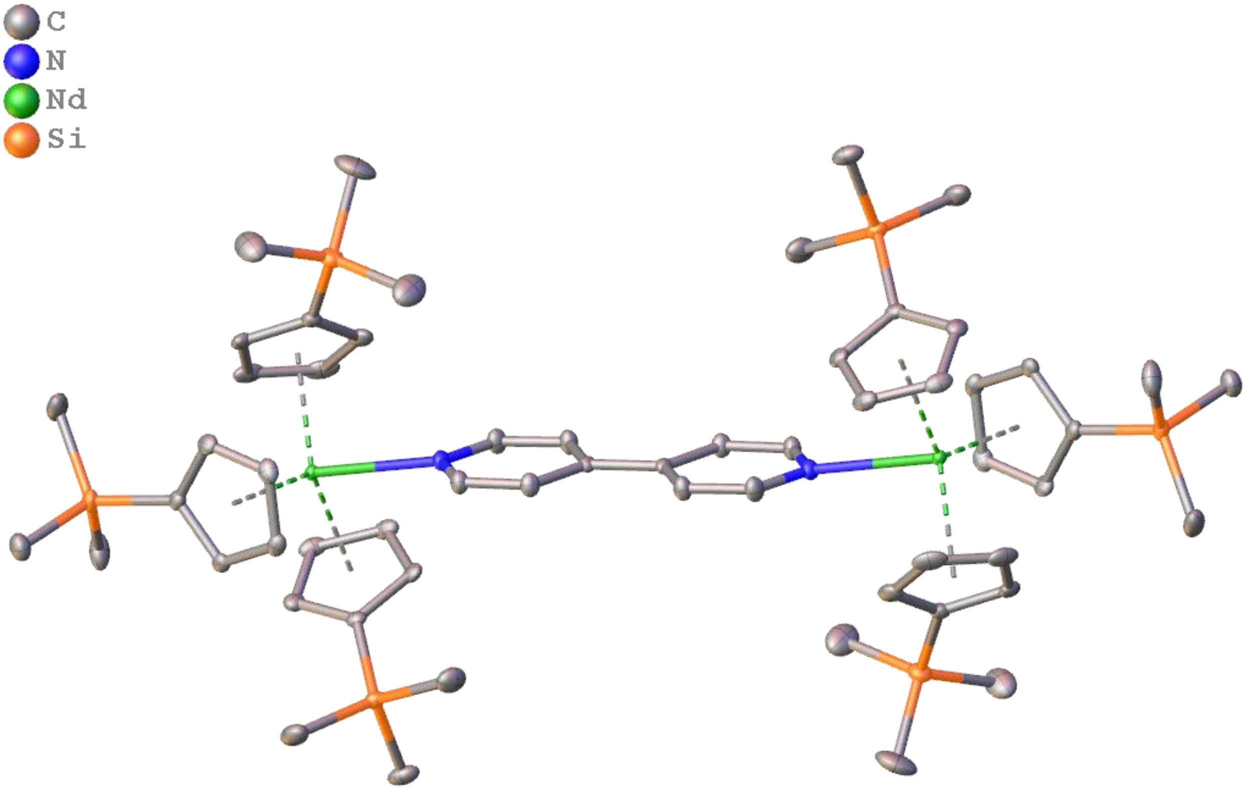

Supplementary Figure 4. Structure of $(Cp'_3Nd)_2(\mu{-}4,4'{-}bpy)$, **1−Nd**, modeled with thermal ellipsoids at 50% probability. Hydrogen omitted for clarity.

Supplementary Table 2: Crystal data and structure refinement for **1−Nd.**

Deposition Number	2081956
Empirical formula	$C_{58}H_{86}N_2Nd_2Si_6$
Formula weight (g/mol)	1268.30
Temperature/K	120
Crystal system	triclinic
Space group	$P\bar{1}$
a/Å	9.5008(12)
b/Å	11.4116(15)
c/Å	14.8923(19)
α/°	99.281(2)
β/°	101.448(2)
γ/°	95.681(2)
Volume/Å^3	1547.4(3)
Z	1
ρ_{calc} g/cm^3	1.361
μ/mm^{-1}	1.811
F(000)	652.0
Crystal size/mm^3	0.446 × 0.215 × 0.136
Radiation	Mo K_α (λ = 0.71073 Å)
2Θ range for data collection/°	4.7 to 55.408
Index ranges	$-12 \le h \le 12$, $-14 \le k \le 14$, $-19 \le l \le 19$
Reflections collected	24169
Independent reflections	7231 [R_{int} = 0.0277, R_{sigma} = 0.0271]
Data/restraints/parameters	7231/0/316
Goodness-of-fit on F^2	1.059
Final R indexes [I ≥ 2σ (I)]	R_1 = 0.0209, wR_2 = 0.0501
Final R indexes [all data]	R_1 = 0.0236, wR_2 = 0.0513
Largest diff. peak/hole / e Å^{-3}	0.54/−1.01

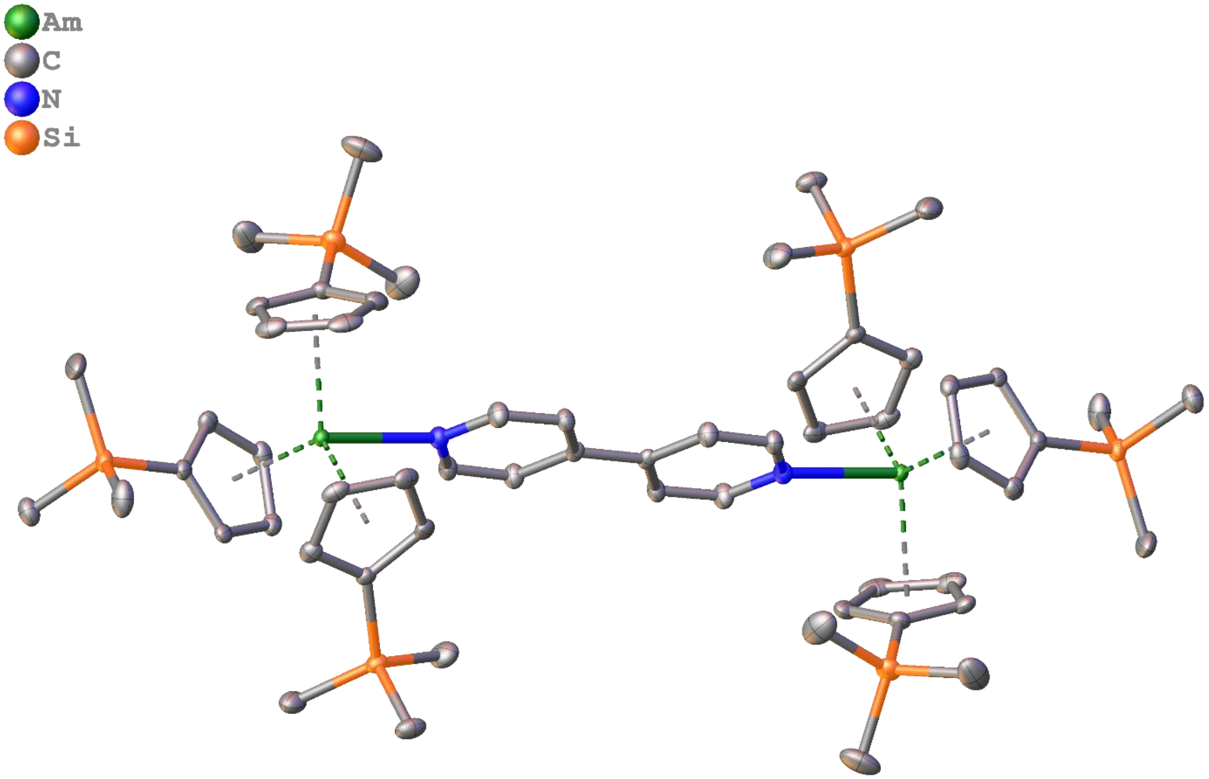

Supplementary Figure 5. Structure of (Cp′$_3$Am)$_2$(μ−4,4′−bpy), **1−Am**, modeled with thermal ellipsoids at 50% probability. Hydrogen omitted for clarity.

Supplementary Table 3. Crystal data and structure refinement for **1−Am**.

Deposition Number	2081957
Empirical formula	$C_{58}H_{86}Am_2N_2Si_6$
Formula weight (g/mol)	1465.82
Temperature/K	100
Crystal system	triclinic
Space group	$P\bar{1}$
a/Å	9.4963(16)
b/Å	11.375(2)
c/Å	14.818(3)
α/°	99.300(5)
β/°	101.542(5)
γ/°	95.603(5)
Volume/Å^3	1533.6(5)
Z	1
ρ_{calc} g/cm^3	1.587
μ/mm^{-1}	2.636
F(000)	722.0
Crystal size/mm^3	0.712 × 0.269 × 0.186
Radiation	Mo K_{α} (λ = 0.71073 Å)
2Θ range for data collection/°	4.204 to 55.106
Index ranges	$-12 \leq h \leq 12$, $-14 \leq k \leq 14$, $-19 \leq l \leq 19$
Reflections collected	69703
Independent reflections	7030 [R_{int} = 0.0538, R_{sigma} = 0.0239]
Data/restraints/parameters	7030/0/316
Goodness-of-fit on F^2	1.289
Final R indexes [I ≥ 2σ (I)]	R_1 = 0.0160, wR_2 = 0.0536
Final R indexes [all data]	R_1 = 0.0181, wR_2 = 0.0678
Largest diff. peak/hole / e Å^{-3}	1.22/−1.16

Supplementary Table 4. Bond lengths and bond angles observed in **1−Nd** and **1−Am**, as well as reported values of similar systems reported for **Ce**, **Ce(py)**, **Nd,** **Th**, and **U**. **Ce = [(MeC$_5$H$_4$)$_3$Ce]$_2$(4,4′−bpy)**,[15] **Ce(py) = Cp′$_3$Ce(py)**,[16] **Nd = Cp$_3$Nd(py)**,[17] **Th = (Cp′′$_3$Th)$_2$(*μ*−4,4′−bpy)**,[18] and **U = (Cp′$_3$U)$_2$(*μ*−4,4′−bpy)**.[5]

**Bond**	**Length (Å)**						
**Compound**	**Ce**	**Ce(py)**	**1−Nd**	**Nd**	**Th**	**U**	**1−Am**
**M1−N1**	**2.673(5)**	**2.704(4)**	**2.6482(16)**	**2.668(5)**	**2.362(4)**	**2.626(7)**	**2.618(2)**
**M1−Cent1**	2.54	2.573(5)	2.535(2)	2.548(13)	2.606(5)	2.551(10)	2.544(3)
**M1−Cent2**	2.56	2.574(5)	2.561(2)	2.556(15)	2.578(5)	2.521(10)	2.521(3)
**M1−Cent3**	2.58	2.587(5)	2.542(2)	2.552(15)	2.597(5)	2.549(10)	2.506(3)
**M1−C1**	2.898(6)	2.843(5)	2.8000(19)	2.819(10)	2.881(5)	2.902(9)	2.899(3)
**M1−C2**	2.819(6)	2.830(5)	2.8338(19)	2.826(8)	2.912(5)	2.799(9)	2.818(3)
**M1−C3**	2.751(6)	2.823(5)	2.8093(19)	2.824(7)	2.923(5)	2.759(9)	2.739(3)
**M1−C4**	2.751(7)	2.849(5)	2.7751(18)	2.779(9)	2.831(5)	2.774(9)	2.768(3)
**M1−C5**	2.832(7)	2.843(5)	2.7722(18)	2.781(9)	2.800(5)	2.865(9)	2.844(3)
**M1−C9**	2.872(6)	2.847(5)	1.8660(19)	2.796(12)	2.815(4)	2.798(9)	2.813(3)
**M1−C10**	2.818(6)	2.863(5)	2.8296(19)	2.792(15)	2.815(4)	2.763(9)	2.831(3)
**M1−C11**	2.782(6)	2.851(5)	2.7621(19)	2.796(12)	2.877(4)	2.780(9)	2.811(3)
**M1−C12**	2.809(6)	2.816(5)	2.7877(19)	2.812(12)	2.872(4)	2.791(8)	2.744(3)
**M1−C13**	2.868(7)	2.825(5)	2.860(2)	2.809(12)	2.843(4)	2.840(9)	2.763(3)
**M1−C17**	2.852(6)	2.924(5)	2.835(2)	2.797(8)	2.895(5)	2.843(10)	2.782(3)
**M1−C18**	2.884(6)	2.853(5)	2.7890(19)	2.836(12)	2.880(5)	2.785(9)	2.818(3)
**M1−C19**	2.870(7)	2.797(5)	2.761(2)	2.834(12)	2.893(5)	2.775(9)	2.794(3)
**M1−C20**	2.826(6)	2.807(5)	2.827(2)	2.792(9)	2.833(4)	2.843(9)	2.753(3)
**M1−C21**	2.815(6)	2.868(5)	2.847(2)	2.778(8)	2.813(4)	2.851(9)	2.751(3)
**N1−C25**	1.357(8)	1.362(6)	1.349(3)	-	1.393(6)	1.339(11)	1.344(4)
**C25−C26**	1.380(8)	1.362(8)	1.383(3)	-	1.328(6)	1.365(12)	1.386(4)
**C26−C27**	1.403(8)	1.378(8)	1.397(3)	-	1.441(6)	1.401(12)	1.393(4)
**C27−C27^1**	1.480(11)	-	1.491(4)	-	1.378(9)	1.474(17)	1.489(5)
**C27−C28**	1.394(8)	1.380(8)	1.397(3)	-	1.454(6)	1.388(12)	1.396(4)
**C28−C29**	1.382(8)	1.378(8)	1.389(3)	-	1.345(6)	1.387(13)	1.380(4)
**C29−N1**	1.339(8)	1.343(6)	1.345(2)	-	1.392(5)	1.364(11)	1.346(4)
**N1−M1−Cent1**	94.9	101.697	103.383	101.2	101.091	93.752	93.655
**N1−M1−Cent2**	99.3	100.110	93.478	93.4	100.480	103.504	101.178
**N1−M1−Cent3**	104.3	96.424	101.405	102.8	102.385	101.178	103.503
**Cent1−M1−Cent2**	117.5	118.297	118.104	117.4	114.233	118.287	116.668
**Cent2−M1−Cent3**	115.3	119.515	116.520	117.3	118.438	117.228	117.408
**Cent3−M1−Cent1**	119.0	114.307	117.436	117.8	116.037	116.463	117.949

**M1−Cent$_{avg}$**	2.56	2.578(5)	2.546(2)	2.552(15)	2.594(5)	2.540(10)	2.524(3)
**M1−C$_{avg}$**	2.830(7)	2.843(5)	2.813(2)	2.805(11)	2.859(5)	2.811(10)	2.795(3)
**Cent−M1−Cent avg**	117.267	117.373	117.353	117.5	116.236	117.326	117.342

Supplementary Note 4: Solid-State UV-vis-NIR Spectra

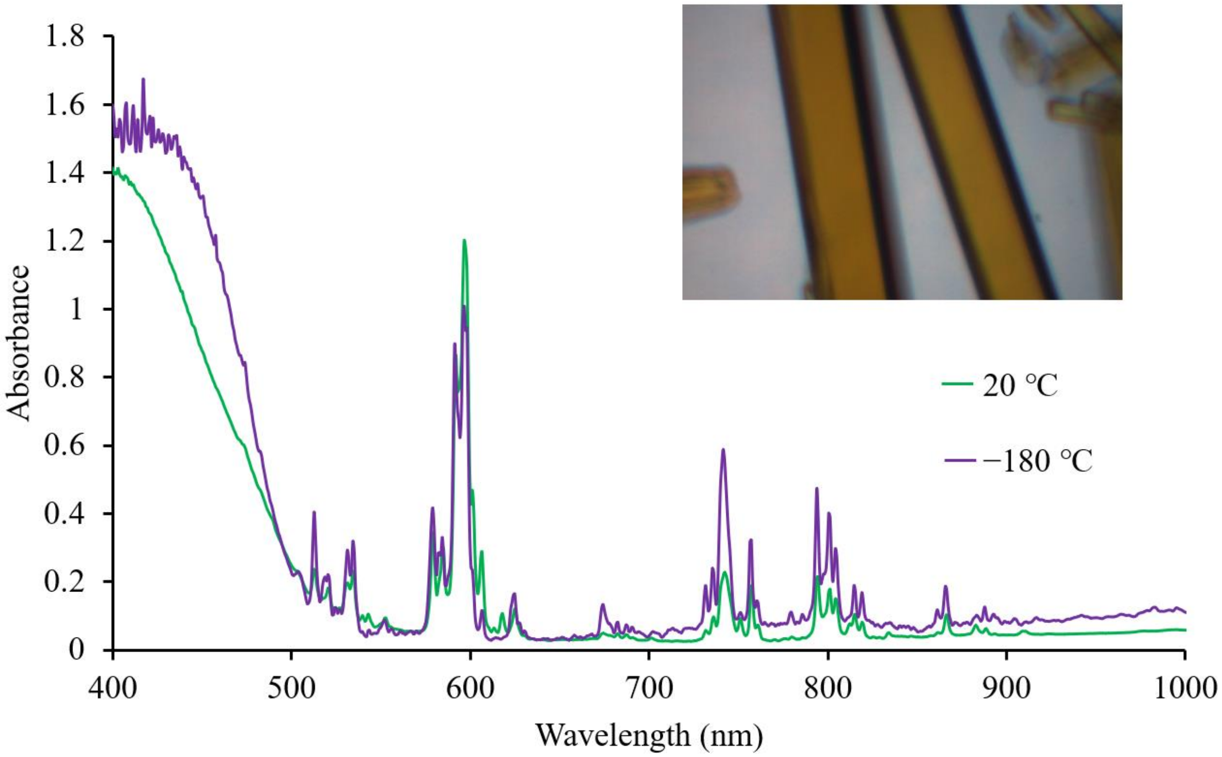

Supplementary Figure 6. Solid-state UV-vis-NIR spectra of **1−Nd** at 20°C (green) and −180 °C (purple).

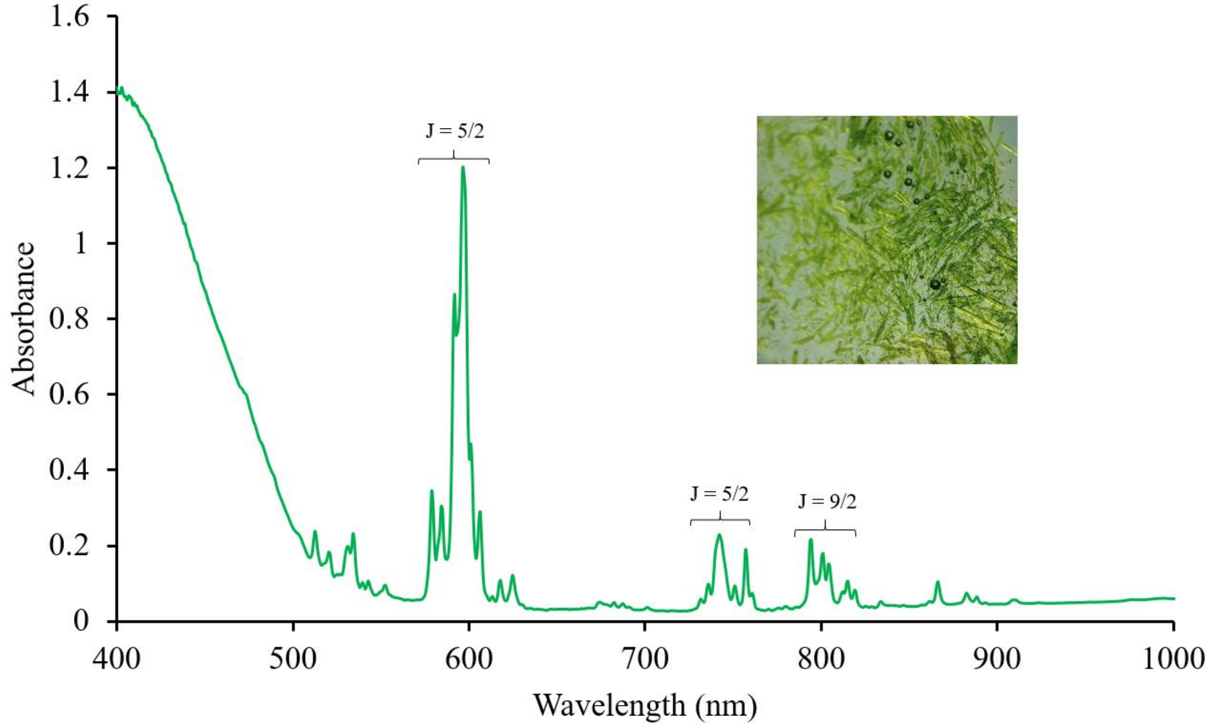

Supplementary Figure 7. Spin-orbit CASSCF/MC-pDFT (SO-pDFT) states of **1−Nd** represented in terms of total angular momentum, *J*.

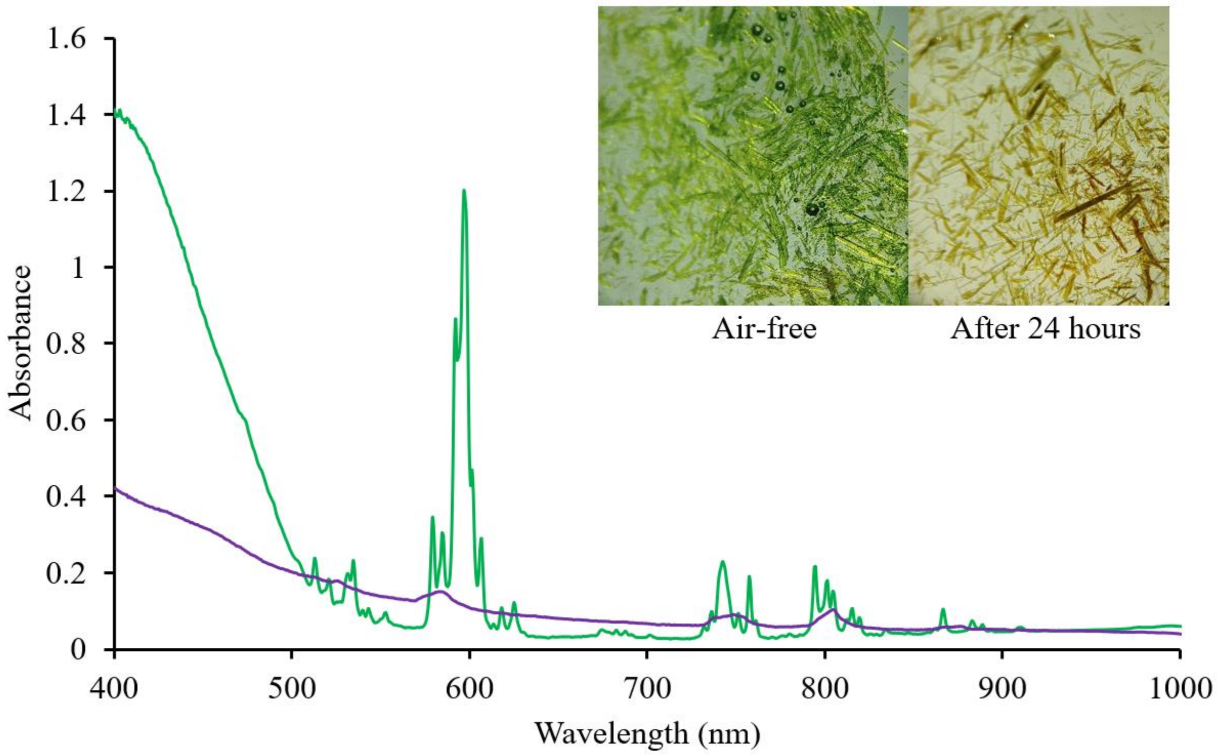

Supplementary Figure 8. Solid-state UV-vis-NIR spectra of **1−Nd** before (green) and after (purple) 24 hours of air exposure.

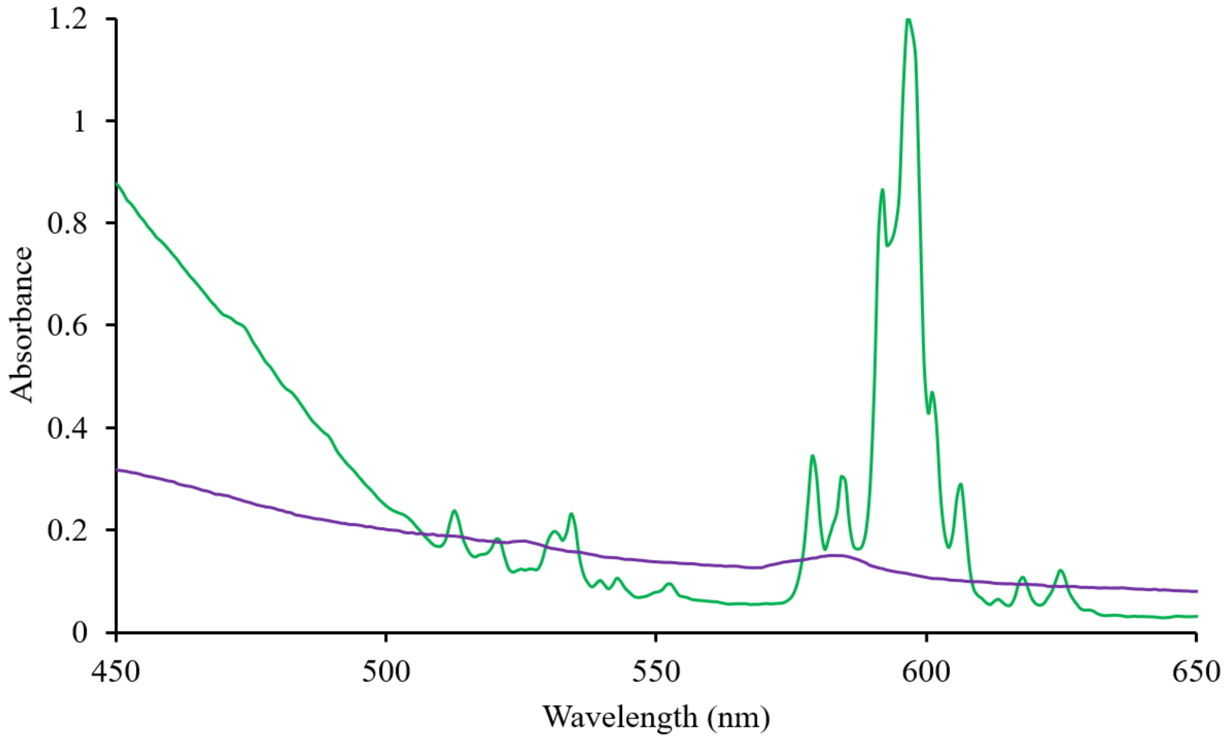

Supplementary Figure 9. Shift and decreased intensity of the hypersensitive 597 nm transition in the solid-state UV-vis-NIR spectra of **1–Nd** before (green) and after (purple) 24 hours of air exposure.

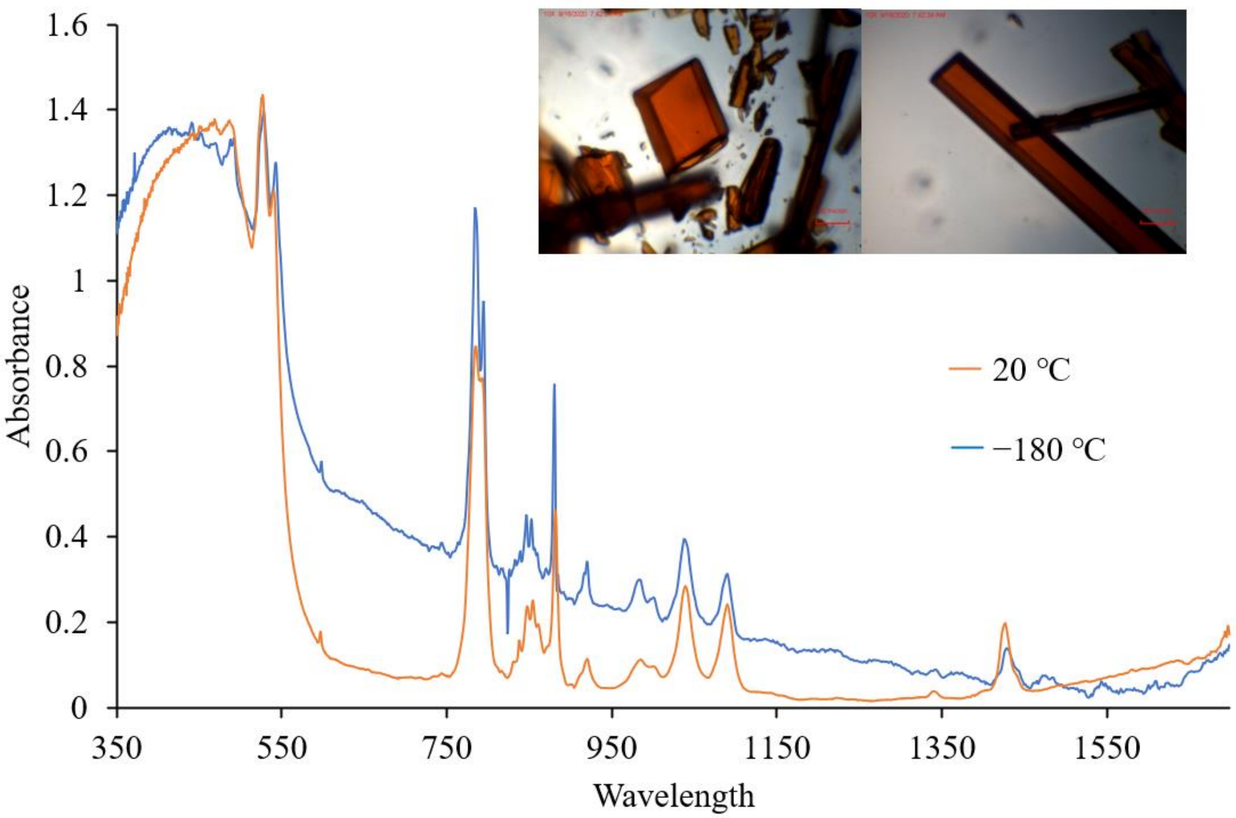

Supplementary Figure 10. Solid-state UV-vis-NIR spectra of **1−Am** at 20°C (green) and −180 °C (purple). **Top right**: Crystals of **1−Am** used to collect spectrum.

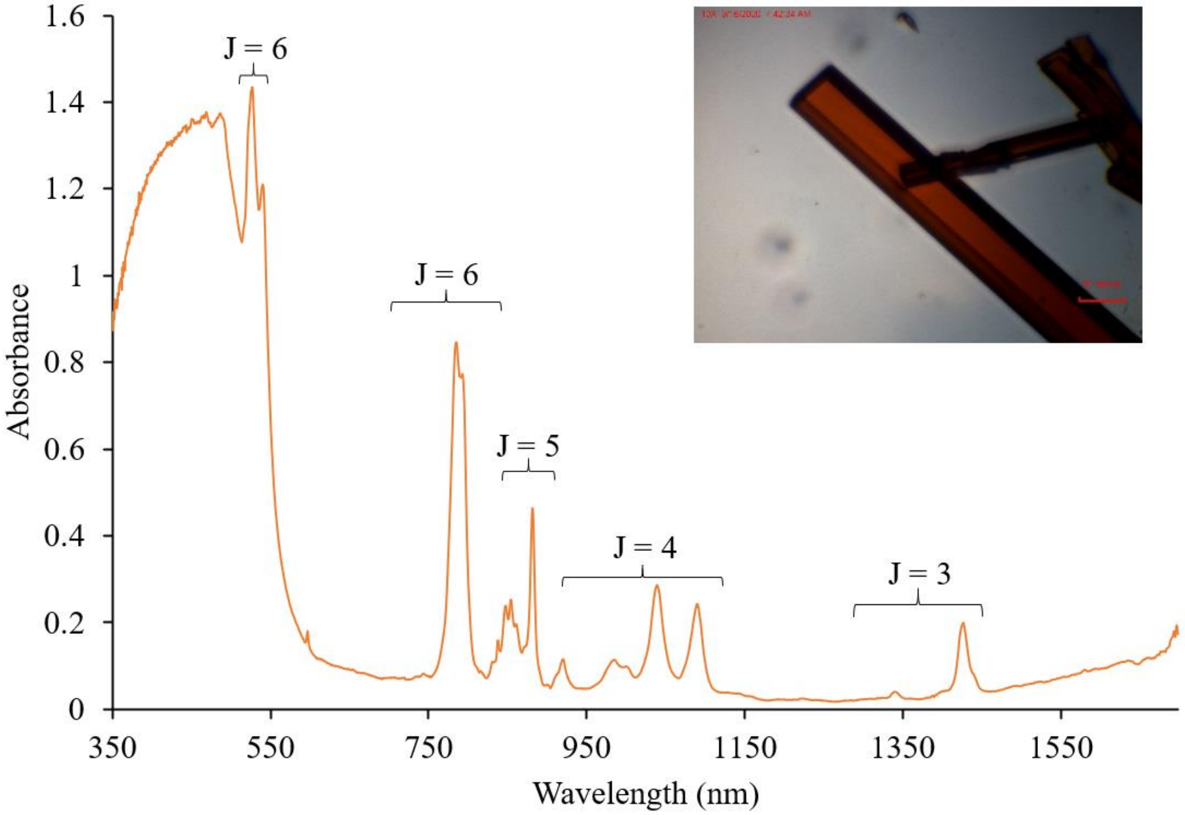

Supplementary Figure 11. Spin-orbit CASSCF/MC-pDFT (SO-pDFT) states of **1−Am** represented in terms of total angular momentum, *J*.

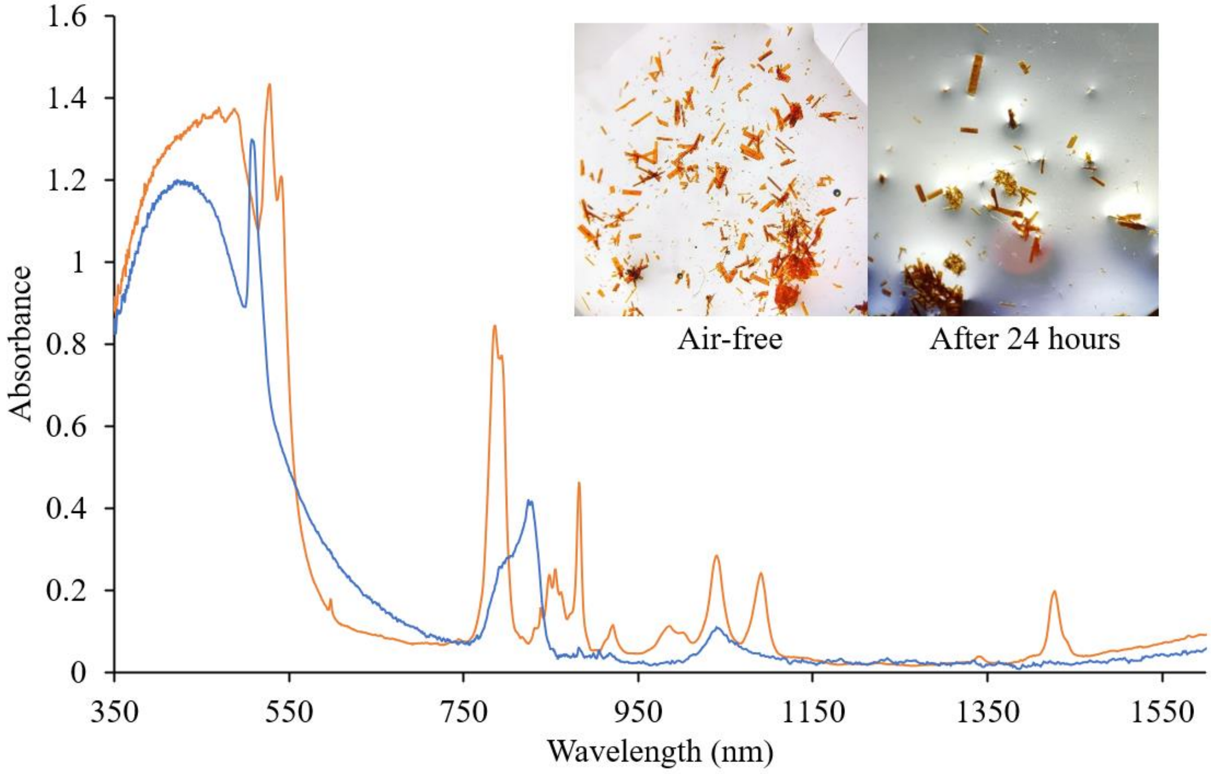

Supplementary Figure 12. Solid-state UV-vis-NIR spectra of **1−Am** before (orange) and after (blue) 24 hours of air exposure.

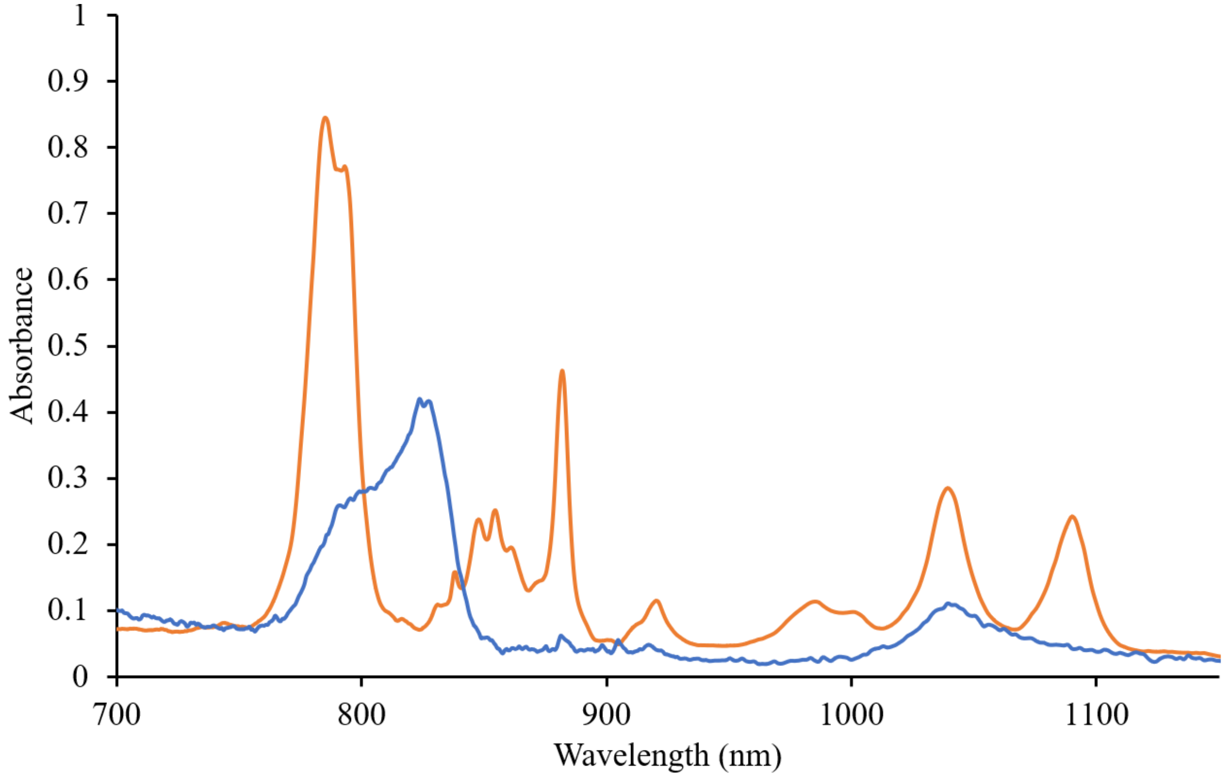

Supplementary Figure 13. Solid-state UV-vis-NIR spectra of **1−Am** before (orange) and after (blue) 24 hours of air exposure, showing a significant decrease in transition splitting upon crystal degradation.

Supplementary Note 5: Solution Phase UV-vis-NIR Spectra

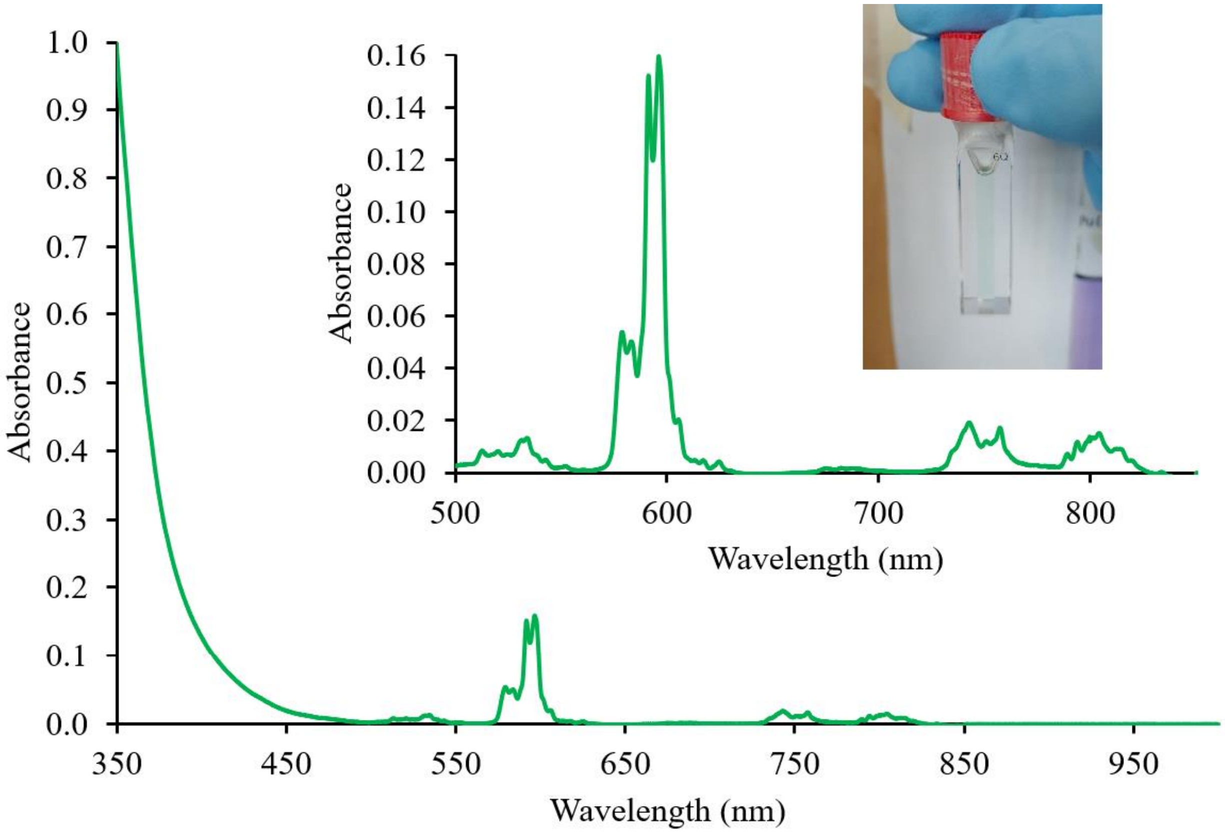

Supplementary Figure 14. Solution phase UV-vis-NIR spectrum of **1−Nd** at room temperature.

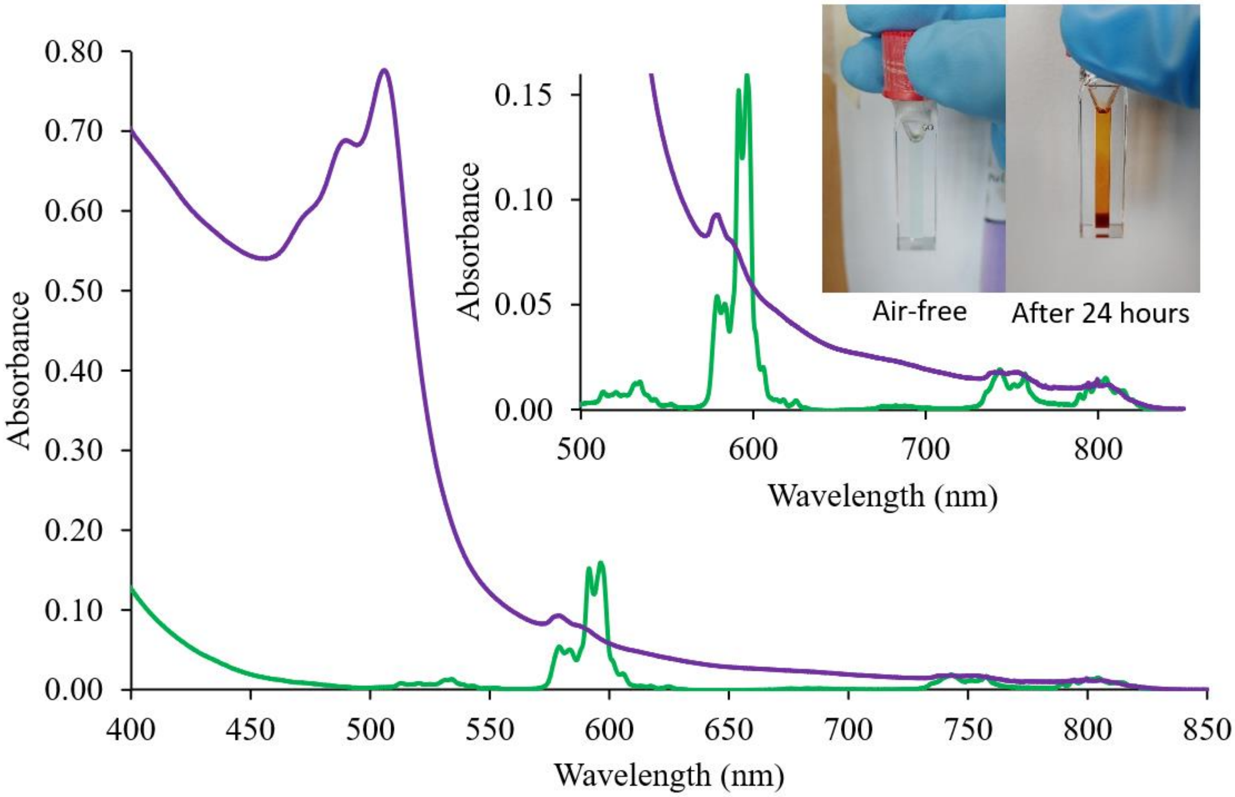

Supplementary Figure 15. Solution phase UV-vis-NIR spectra of **1−Nd** before (green) and after (purple) 24 hours of air exposure. A broad transition from 450 nm to 550 nm is observed following air exposure of the sample. **Top right**: Formation of a brown precipitate and a light brown/orange solution is seen after **1−Nd** is exposed to air.

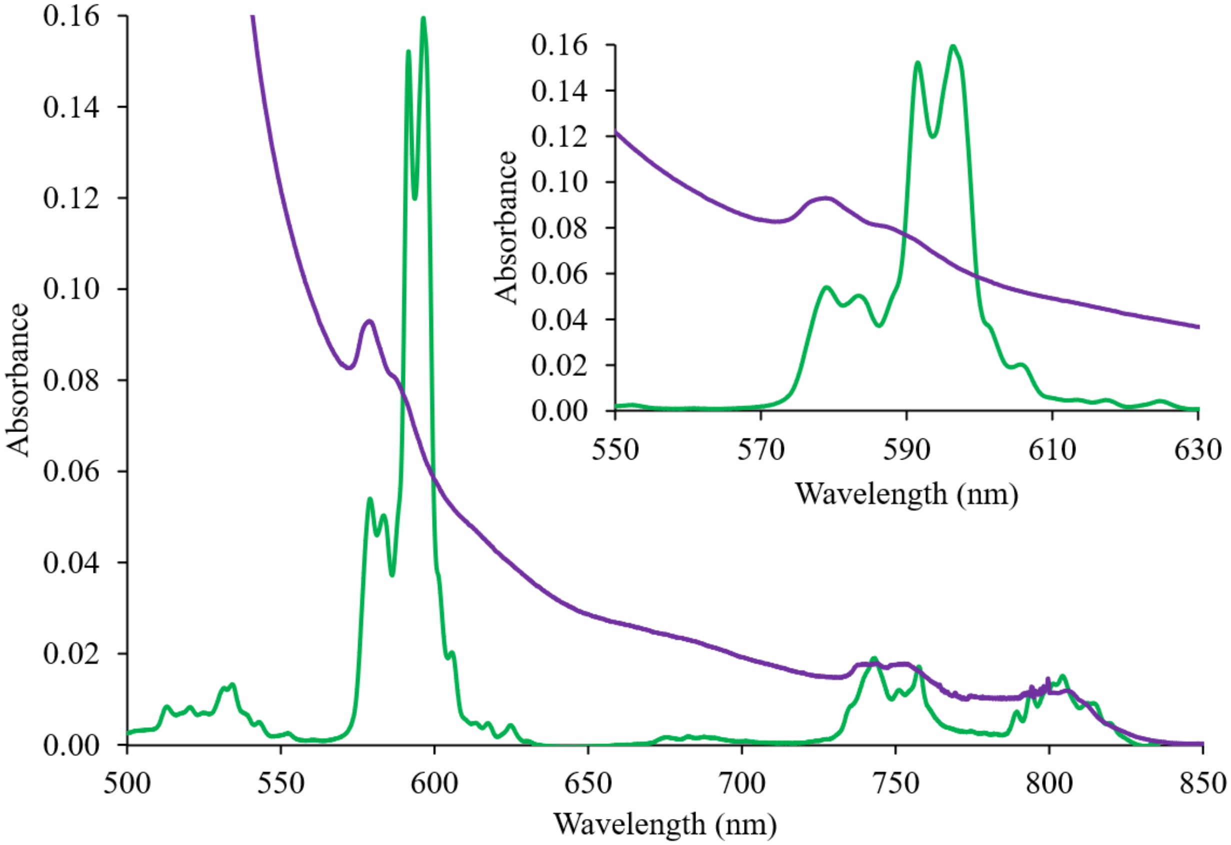

Supplementary Figure 16. Shift and decreased intensity of the hypersensitive 591 nm and 597 nm transition in the solution phase UV-vis-NIR spectra of **1−Nd** before (green) and after (purple) 24 hours of air exposure. Significant splitting is no longer observed.

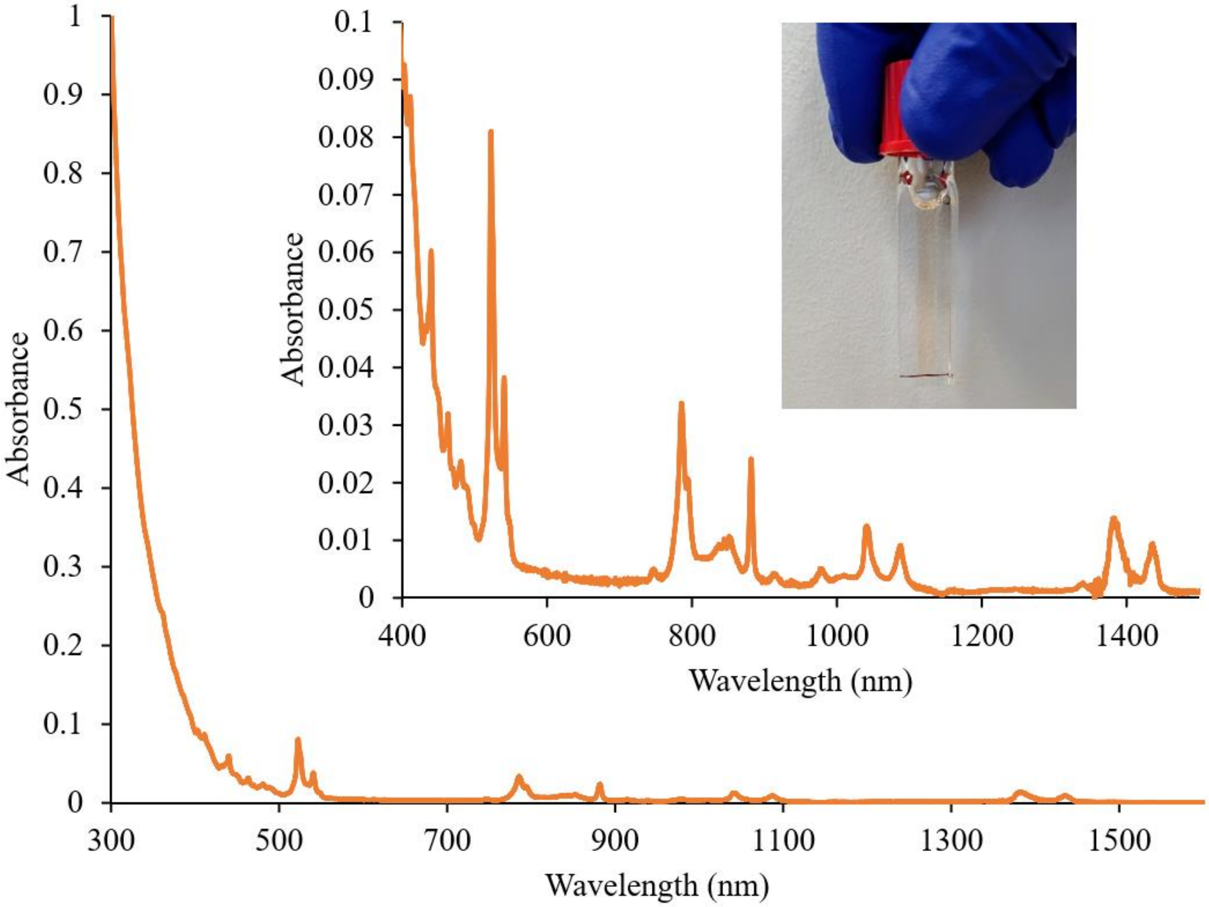

Supplementary Figure 17. Solution phase UV-vis-NIR spectra of **1−Am** at room temperature.

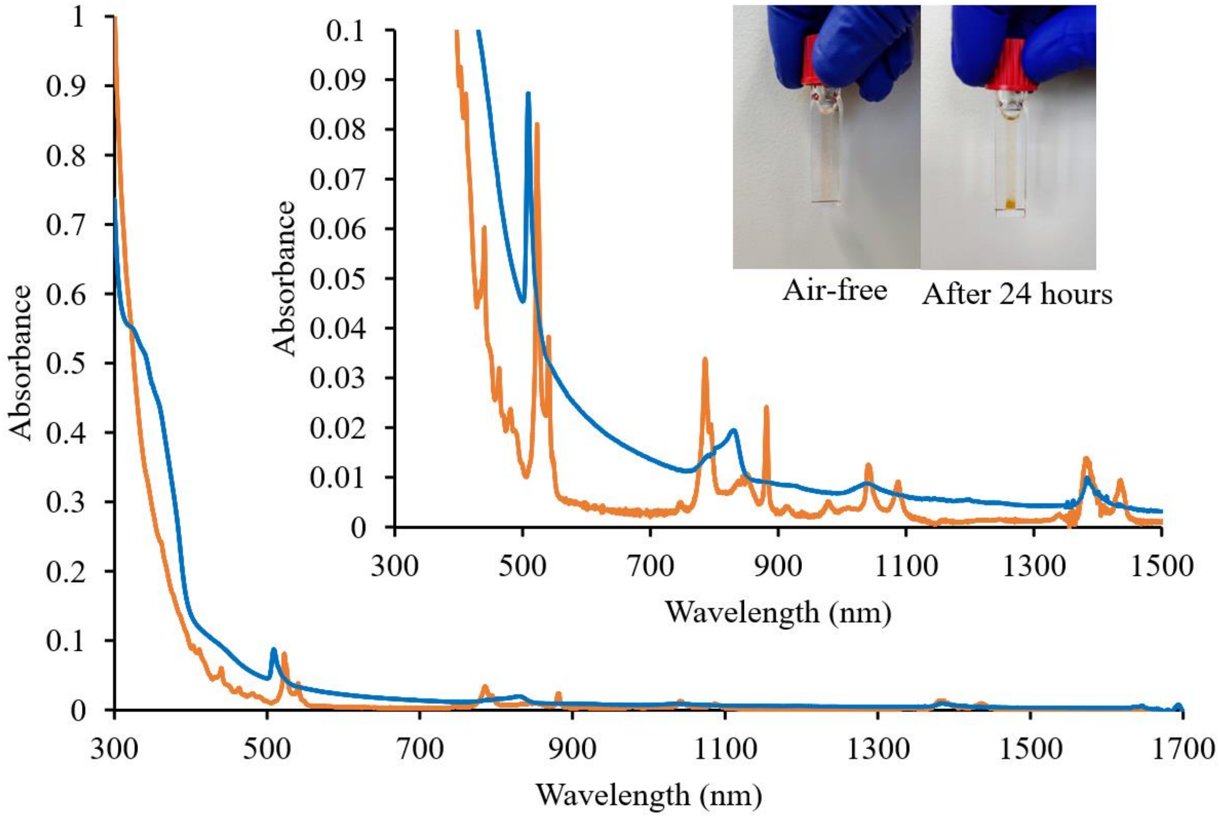

Supplementary Figure 18. Solution phase UV-vis-NIR spectra of **1−Am** before (orange) and after (blue) 24 hours of air exposure. **Top right**: A brown precipitate forms upon air exposure resulting in a light orange/pink solution.

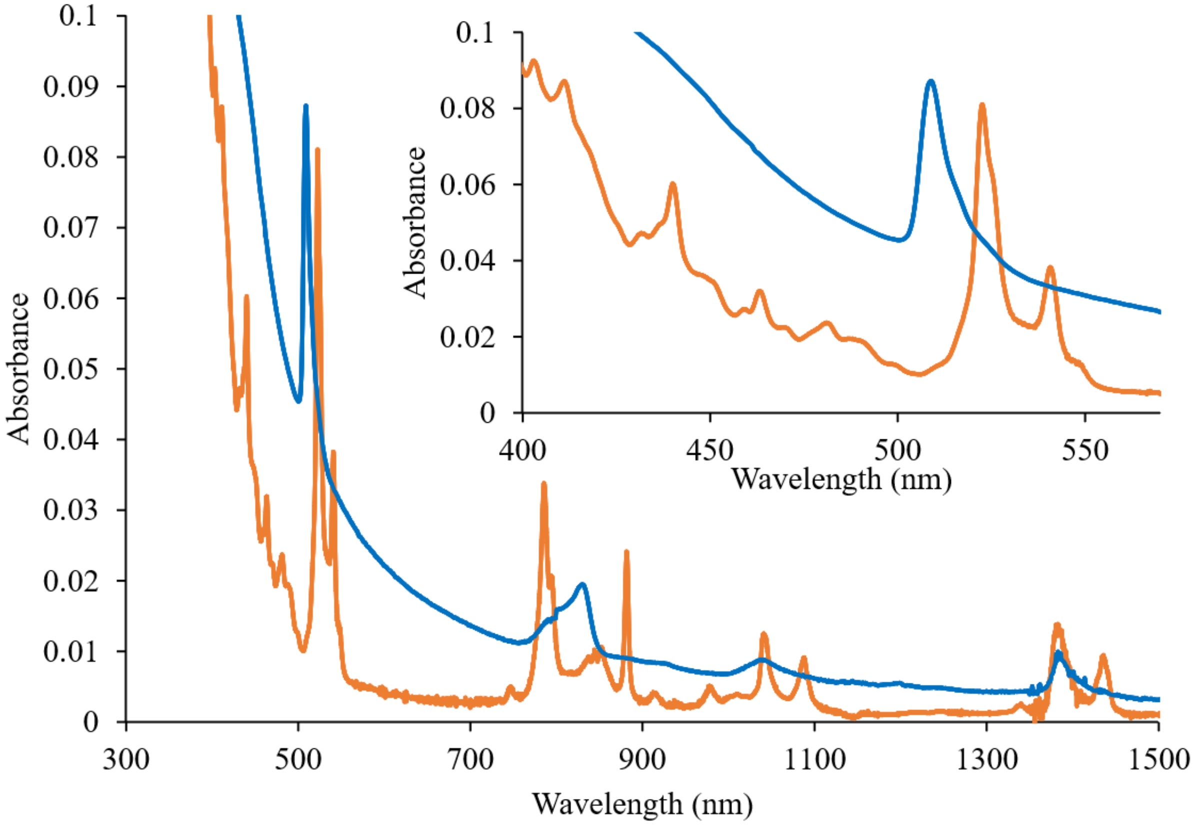

Supplementary Figure 19. Solution phase UV-vis-NIR spectra of **1−Am** before (orange) and after (blue) 24 hours of air exposure. A decrease in splitting is observed, as well as a shift to higher energy of the hypersensitive 522 nm transition upon the decomposition of **1−Am**.

Supplementary Note 6: ^{1}H NMR Spectra

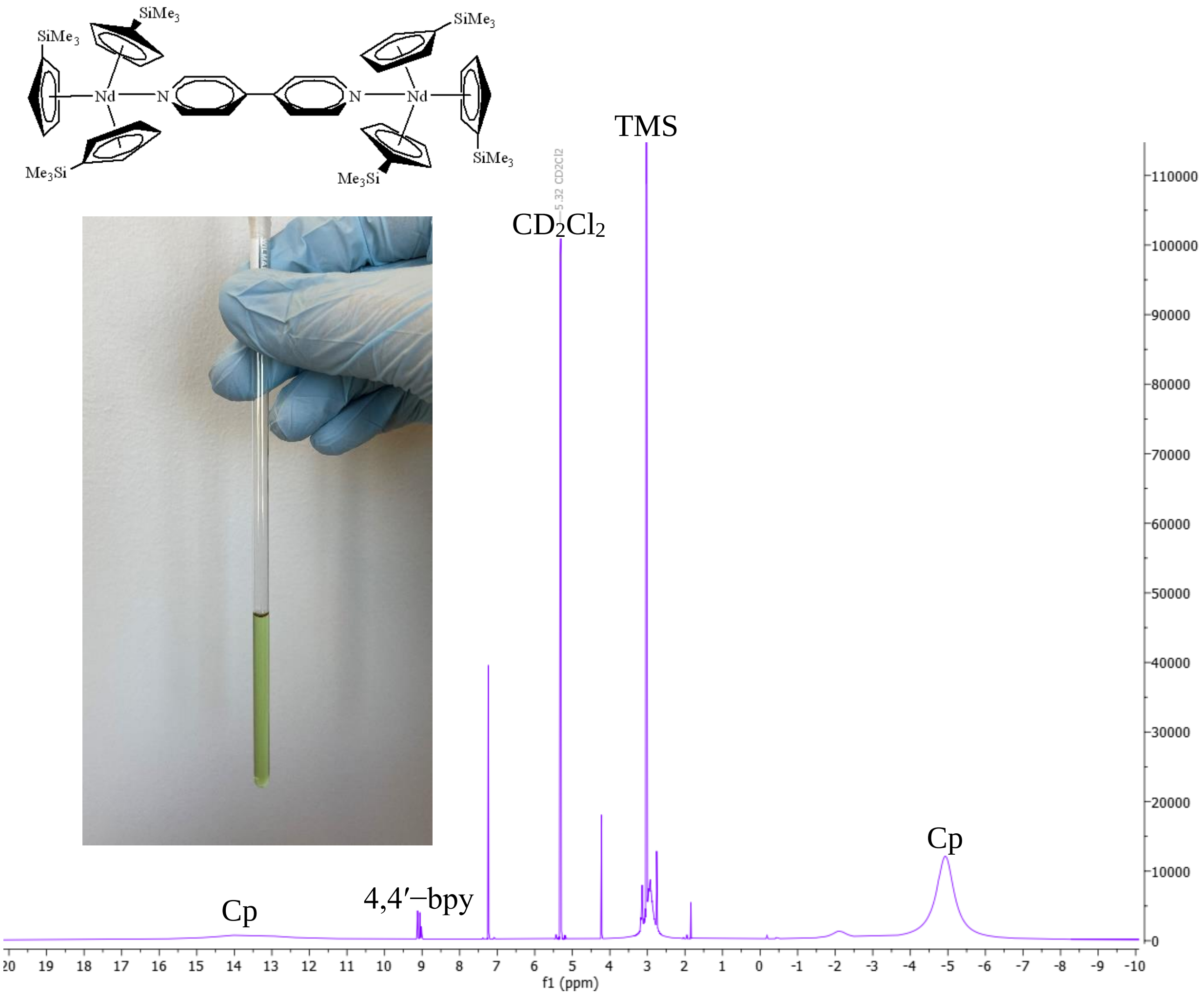

Supplementary Figure 20. ^{1}H NMR spectrum of **1−Nd** in CD_2Cl_2 at 298 K. Significant broadening of Cp peaks is observed

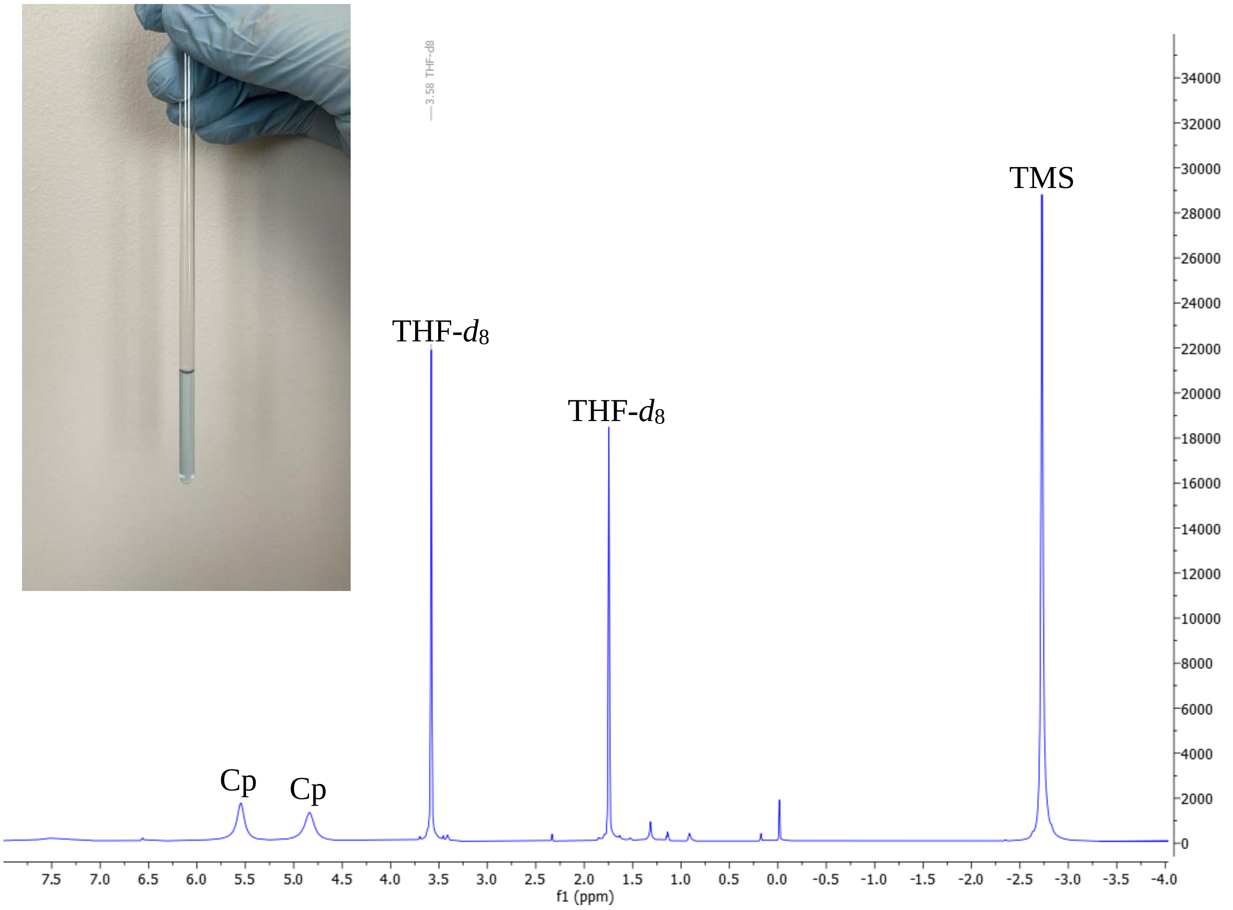

Supplementary Figure 21. ^{1}H NMR spectrum of **1–Nd** in THF-d_8 at 298 K. A shift in the Cp peaks is observed and the weakly coordinated 4,4′–bpy is likely replaced by the coordination of the solvent.

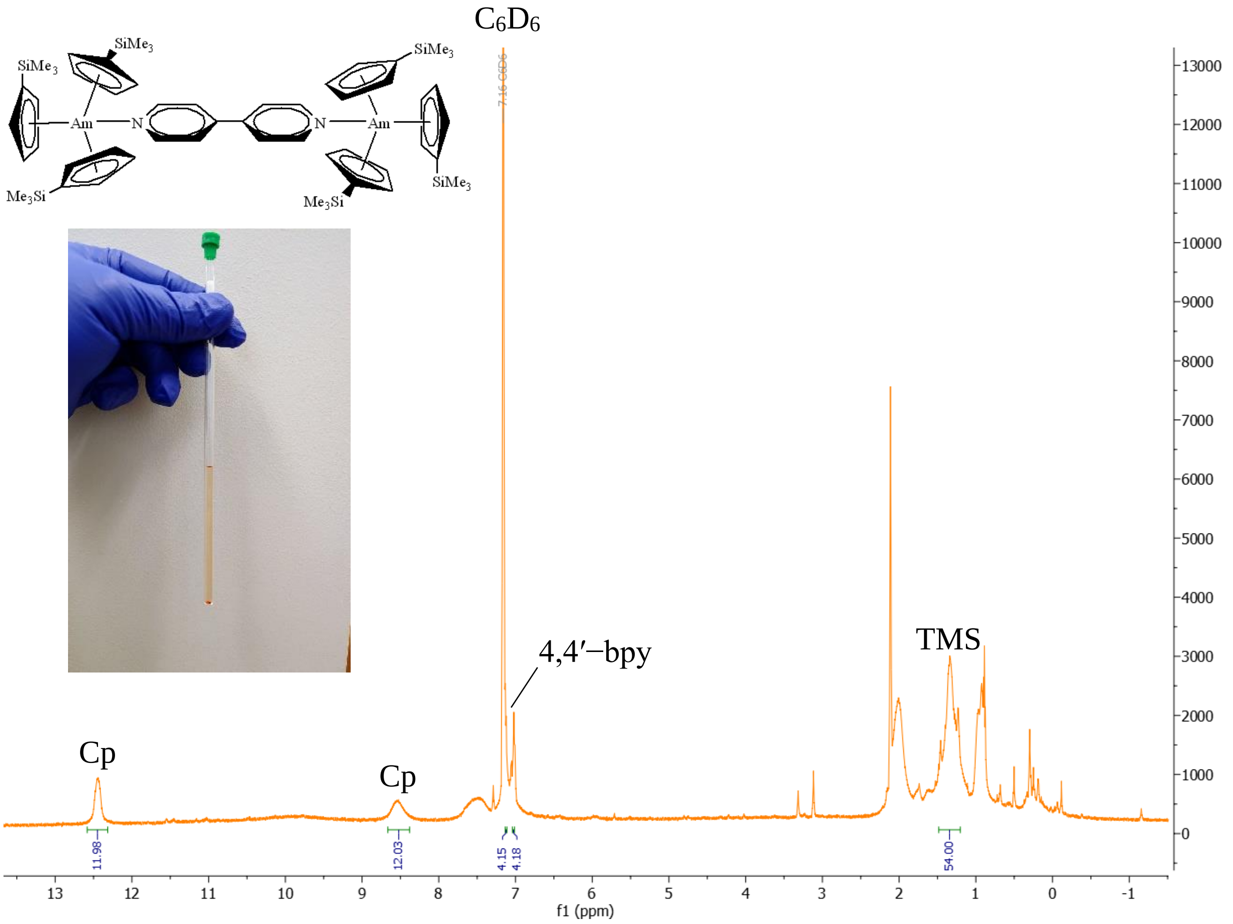

Supplementary Figure 22. ^{1}H NMR spectrum of **1−Am** in C_6D_6 at 298 K.

Supplementary Note 7: Magnetic Susceptibility

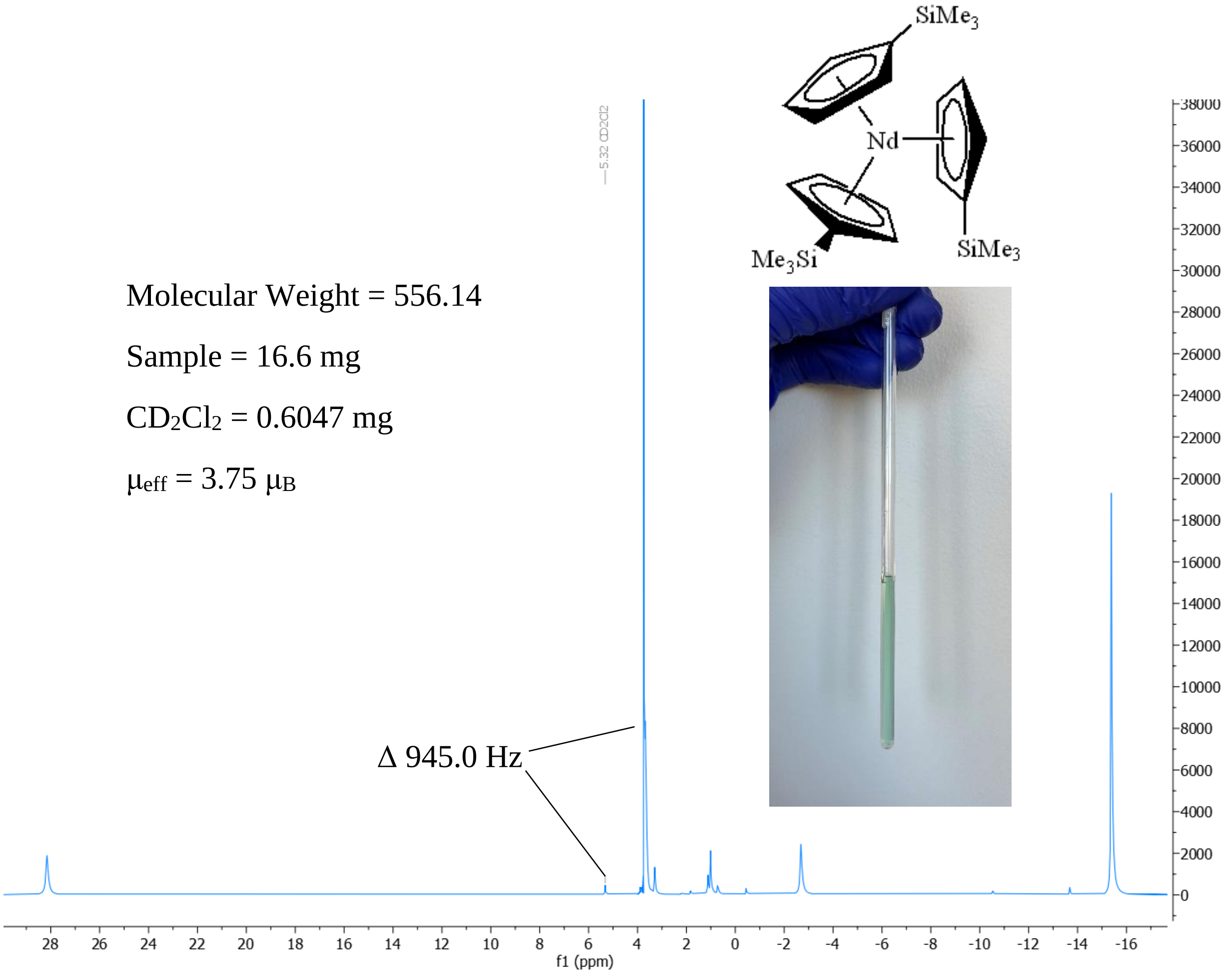

Supplementary Figure 23. ^{1}H NMR Evans Method measurements of Cp'_3Nd in CD_2Cl_2 at 293.4 K.

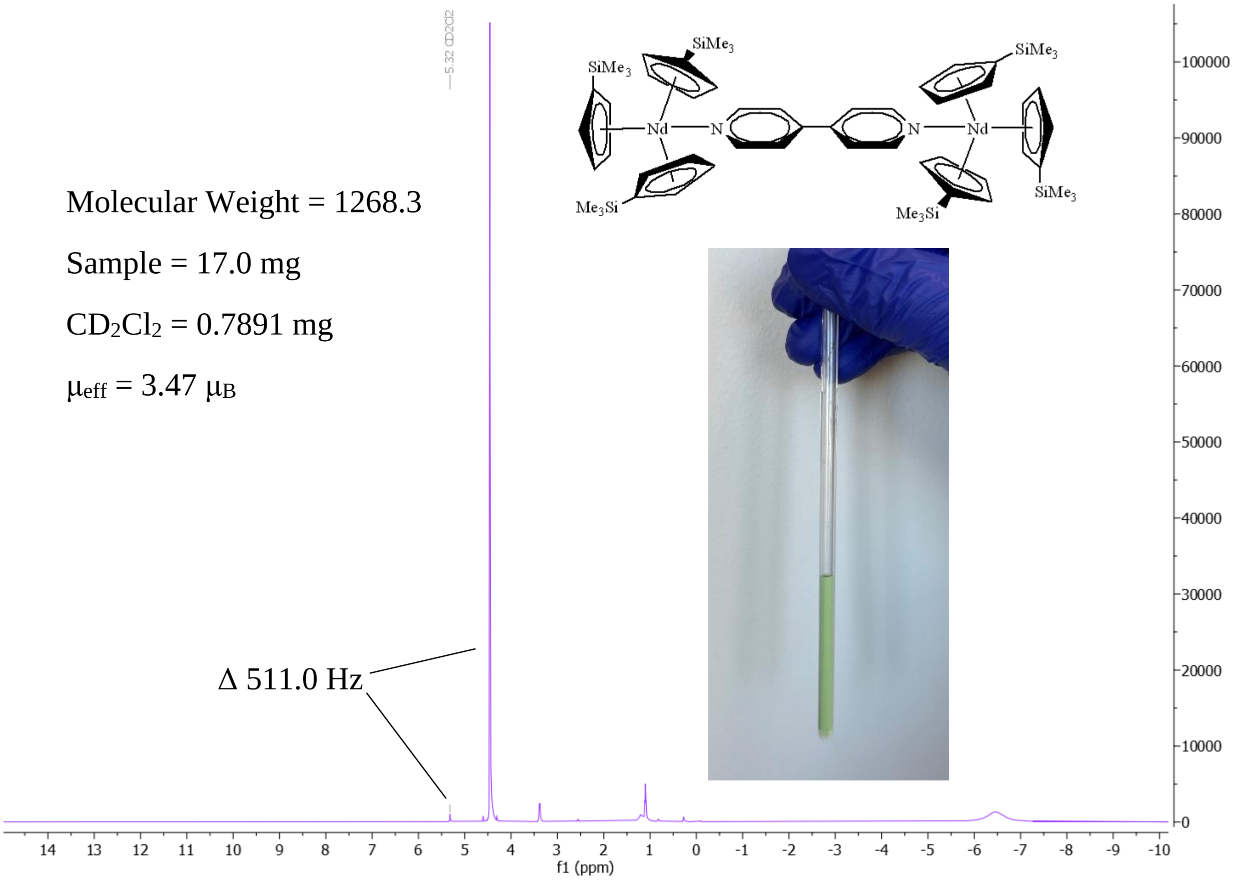

Supplementary Figure 24. ^{1}H NMR Evans Method measurements of 1−Nd in CD_2Cl_2 at 293.4 K.

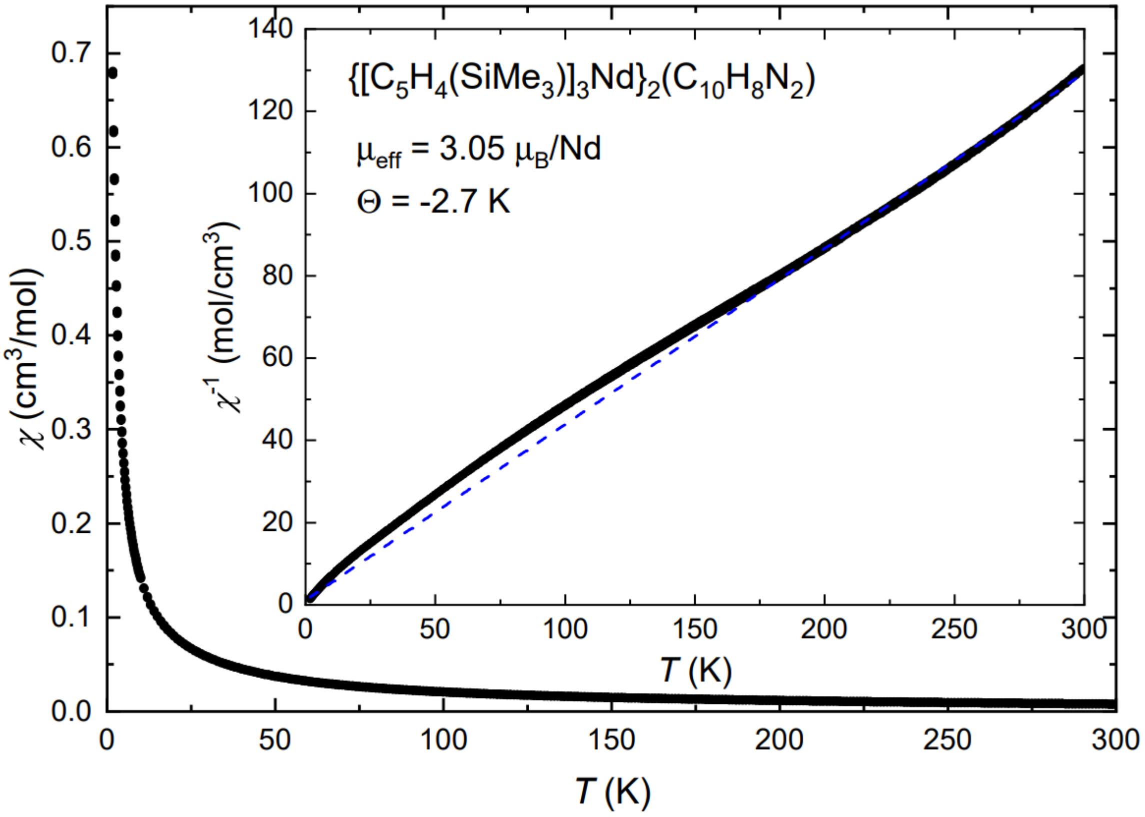

Supplementary Figure 25. Variable temperature magnetic susceptibility measurements of **1−Nd** from T = 1.8 – 300 K. Measurements were taken with an applied magnetic field of H = 5 kOe.

Supplementary Note 8: SO-pDFT and QTAIM

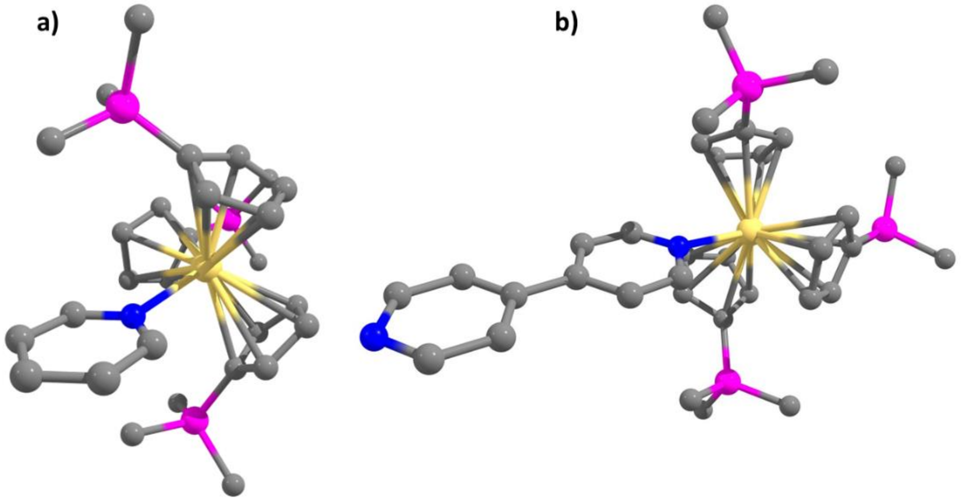

Supplementary Figure 26. Models used to perform the calculations a) $(Cp'_3M)_2(py)$, Mod1 b) $(Cp'_3M)_2(\mu-4,4'-bpy)$, Mod2 (M = Nd^{3+}, U^{3+}, and Am^{3+}). Metal ions, carbon, nitrogen, and silicon are depicted in pale yellow, gray, blue and magenta, respectively. Hydrogen atoms are omitted for clarity.

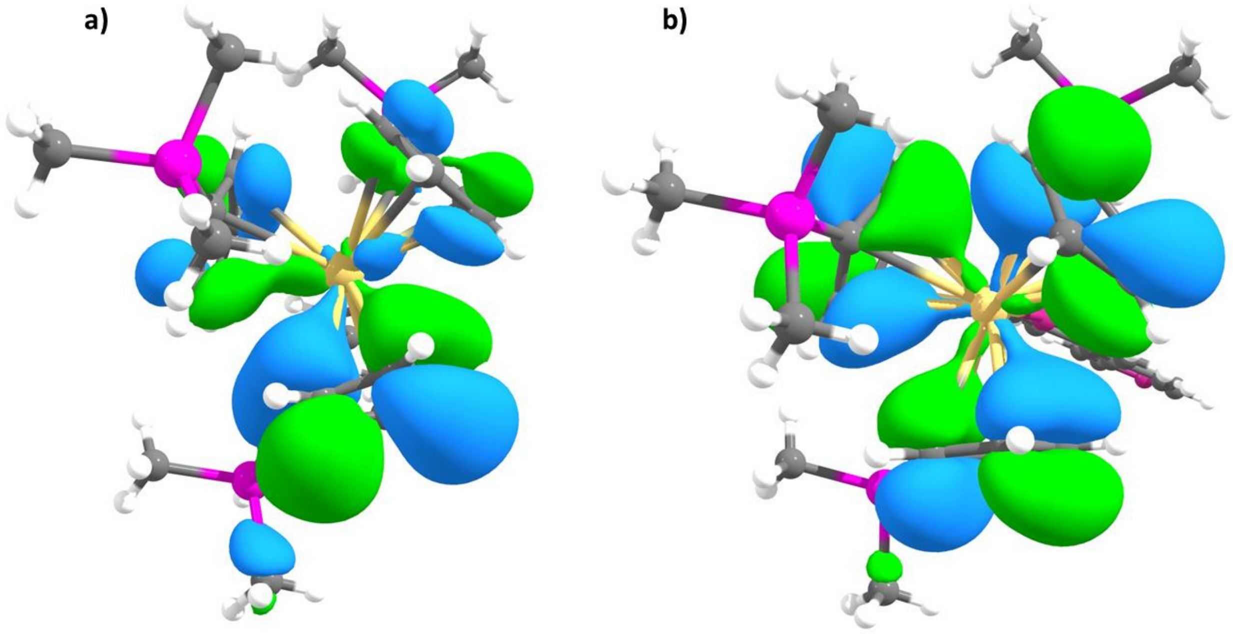

Supplementary Figure 27. Depiction of the natural active molecular orbitals with bonding character between π orbitals from Cp′ rings and the $f_{y(y^2-3x^2)}$ orbital from the metal in **a) 1−Am**, **b) 1−Nd**. Isosurfaces at ±0.025 au.

Supplementary Table 5. Assignment of the SO-CAS and SO-pDFT states for **1−Am** in terms of the total angular momentum quantum number, J. The most predominant Russell-Saunders term is also indicated in the second column. For the sake of clarity, the barycenters of energy are shown.

J	Russell-Saunders Term	SO-CAS Energy (cm^{-1})	SO-pDFT Energy (cm^{-1})	SO-pDFT Wavelength (nm)
0	$^7F_0$	0	0	-
1	$^7F_1$	1970.2	1919.9	5208
2	$^7F_2$	4569.1	4210.7	2375
3	$^7F_3$	7206.3	6239.2	1603
4	$^7F_4$	9662.1	8298.2	1205
5	$^7F_5$	11436.9	10537.3	949
6	$^7F_6$	12498.7	12335.1	810
0	$^5D_0$	14563.9	15478.6	646
1	$^5D_1$	19361.8	18140	551
6	$^5L_6$	25275.5	19377.8	516

Supplementary Table 6. Assignment of the SO−CAS and SO−pDFT states for **1−Nd** in terms of the total angular momentum quantum number, J. The most predominant Russell-Saunders term is also indicated in the second column. For the sake of clarity, the barycenters of energy are shown.

J	Russell-Saunders Term	SO-CAS Energy (cm^{-1})	SO-pDFT Energy (cm^{-1})	SO-pDFT Wavelength (nm)
9/2	$^4I_{9/2}$	240.4	337.1	29673
11/2	$^4I_{11/2}$	2227.8	2052.1	4873
13/2	$^4I_{13/2}$	4352.9	4005.9	2496
15/2	$^4I_{15/2}$	6600.8	5874.2	1702
3/2	$^4F_{3/2}$	13735.3	11657.1	858
9/2	$^2H_{9/2}$	13945.1	12685.7	788
5/2	$^4F_{5/2}$	14966.7	12888.2	776
3/2, 7/2	$^4S_{3/2}$, $^4F_{7/2}$	15941.5	13789.4	725
9/2	$^4F_{9/2}$	17044.7	14445.8	692
11/2	$^2H_{11/2}$	17213.1	15874.2	630
5/2	$^4G_{5/2}$	19276.2	17164.4	582

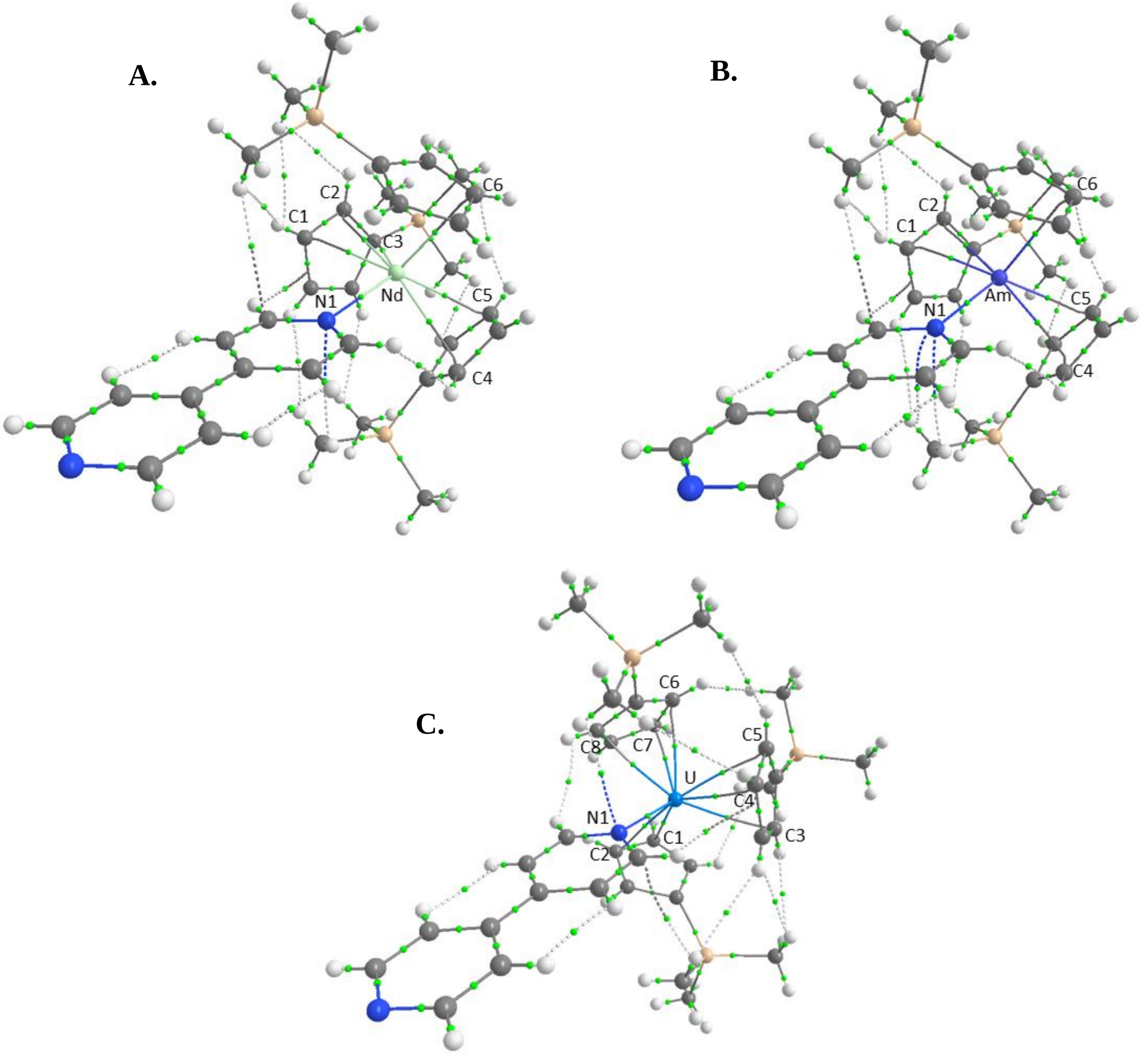

Supplementary Figure 28. QTAIM connectivity for **1−Nd** (**A.**) and **1−U*** (**C.**) from SR-CAS(5,8) and **1−Am** (**B.**) from SR-CAS(8,8) densities.[5] Bond critical points (BCPs) are depicted as green balls. Dashed lines represent bonds where the electron density is below the weak threshold value in AIMAll. The atoms connected to the metal center have been labelled in accordance to Table 1.

Supplementary Table 7. QTAIM metrics of **1−Nd** at the BCP. The electron density, ρ(r), is given in e $Å^{-3}$; whereas potential (V), kinetic (G), and total (H) energy densities in kJ mol−1 $Å^{-3}$. Integrated metrics such as delocalization, δ(r), and localization, λ(M), indices are also provided. An integrated oxidation state, OS, is calculated through the equation OS(M) = Z(M) − λ(M).

Nd−L	ρ(r)	δ(r)	V(r)	G(r)	|V(r)|/G(r)	H(r)	H(r)/ρ(r)	λ(Nd)	OS (Nd)
C1	0.2112	0.1322	−479.0	479.0	1.00	0.0	0.0		
C2	0.2072	0.1267	−477.2	482.5	0.99	5.3	25.4		
C3	0.2038	0.1452	−445.7	449.2	0.99	3.5	17.2		
C4	0.1842	0.1202	−407.1	417.6	0.97	10.5	57.1	57.0	3.0
C5	0.2193	0.1453	−480.8	473.7	1.01	−7.0	−32.0		
C6	0.2166	0.1395	−480.8	475.5	1.01	−5.3	−24.3		
N1	0.2362	0.1831	−554.4	580.8	0.95	26.3	111.4		

Supplementary Table 8. QTAIM metrics of **1−Am** at the BCP. The electron density, ρ(r), is given in e $Å^{-3}$; whereas potential (V), kinetic (G), and total (H) energy densities in kJ mol−1 $Å^{-3}$. Integrated metrics such as delocalization, δ(r), and localization, λ(M), indices are also provided. An integrated oxidation state, OS, is calculated through the equation OS(M) = Z(M) − λ(M).

Am−L	ρ(r)	δ(r)	V(r)	G(r)	|V(r)|/G(r)	H(r)	H(r)/ρ(r)	λ(Am)	OS (Am)
C1	0.2328	0.1548	−610.6	584.3	1.05	−26.3	−113.0		
C2	0.2335	0.1502	−605.3	577.3	1.05	−28.1	−120.2		
C4	0.2011	0.1350	−505.3	498.3	1.01	−7.0	−34.9	92.3	2.7
C5	0.2369	0.1663	−600.1	572.0	1.05	−28.1	−118.5		
C6	0.2301	0.1476	−589.5	568.5	1.04	−21.1	−91.5		
N1	0.2625	0.2260	−759.7	761.5	1.00	1.8	6.7		

Supplementary Table 9. QTAIM metrics of **1−U*** at the BCP.[5] The electron density, ρ(r), is given in e $Å^{-3}$; whereas potential (V), kinetic (G), and total (H) energy densities in kJ mol−1 $Å^{-3}$. Integrated metrics such as delocalization, δ(r), and localization, λ(M), indices are also provided. An integrated oxidation state, OS, is calculated through the equation OS(M) = Z(M) − λ(M).

U*−L	ρ(r)	δ(r)	V(r)	G(r)	|V(r)|/G(r)	H(r)	H(r)/ρ(r)	λ(U*)	OS (U*)
C1	0.2544	0.1917	-614.1	563.2	-1.09	-50.9	-200.0		
C2	0.2483	0.1713	-605.3	559.7	-1.08	-45.6	-183.7		
C3	0.2537	0.1710	-610.6	556.2	-1.10	-54.4	-214.4		
C4	0.2389	0.1883	-579.0	538.7	-1.07	-40.4	-168.9	88.5	3.5
C5	0.2247	0.1567	-531.6	494.8	-1.07	-36.8	-164.0		
C6	0.2477	0.1931	-570.2	510.6	-1.12	-59.7	-240.9		
C7	0.2463	0.2152	-591.3	545.7	-1.08	-45.6	-185.2		
C8	0.2132	0.1614	-496.5	470.2	-1.06	-26.3	-123.4		
N1	0.2807	0.2654	-754.5	736.9	-1.02	-17.5	-62.5		

Supplementary References

1 Dolomanov, O. V., Bourhis, L. J., Gildea, R. J., Howard, J. A. K. & Puschmann, H. *OLEX2*: a complete structure solution, refinement and analysis program. *J. Appl. Crystallogr.* **42**, 339-341 (2009).

2 Sheldrick, G. M. *SHELXT* - Integrated space-group and crystal-structure determination. *Acta Crystallogr. Sect. A. Found. Crystallogr.* **71**, 3-8 (2015).

3 Roos, B. O., Taylor, P. R. & Sigbahn, P. E. M. A Complete Active Space SCF Method (CASSCF) Using a Density Matrix Formulated Super-CI Approach. *Chem. Phys.* **48**, 157-173 (1980).

4 Fdez. Galván, I. *et al.* OpenMolcas: From Source Code to Insight. *J. Chem. Theory. Comput.* **15**, 5925-5964 (2019).

5 Mehdoui, T., Berthet, J. C., Thuery, P. & Ephritikhine, M. CCDC 958634: Experimental Crystal Structure Determination. (2013).

6 Roos, B. O., Lindh, R., Malmqvist, P. A., Veryazov, V. & Widmark, P.-O. Main Group Atoms and Dimers Studied with a New Relativistic ANO Basis Set. *J. phys. Chem. A* **108**, 2851-2858 (2004).

7 Roos, B. O. *et al.* New Relativistic Atomic Natural Orbital Basis Sets for Lanthanide Atoms with Applications to the Ce Diatom and LuF_3. *J. Phys. Chem. A* **112**, 11431-11435 (2008).

8 Hess, B. A. Relativistic Electronic-Structure Calculations Employing a Two-Component No-Pair Formalism with External-Field Projection Operators. *Phys. Rev. A: At., Mol., Opt. Phys.* **33**, 3742 (1986).

9 Dan, D., Celis-Barros, C., White, F. D., Sperling, J. M. & Albrecht-Schmitt, T. E. Origin of selectivity of a triazinyl ligand for americium(III) over neodymium(III). *Chem. Eur. J.* **25**, 3248-3252 (2019).

10 Grimes, T. S. *et al.* Influence of a Heterocyclic Nitrogen-Donor Group on the Coordination of Trivalent Actinides and Lanthanides by Aminopolycarboxylate Complexants. *Inorg. Chem.* **57**, 1373-1385 (2018).

11 Gagliardi, L. *et al.* Multiconfiguration Pair-Density Functional Theory: A New Way To Treat Strongly Correlated Systems. *Acc. Chem. Res.* **50**, 66-73 (2017).

12 Malmqvist, P. A., Roos, B. O. & Schimmelpfennig, B. The Restricted Active Space (RAS) State Interaction Approach with Spin-Orbit Coupling. *Chem. Phys. Lett.* **357**, 230-240 (2002).

13 Bader, R. *Atoms in Molecules*. (Oxford University Press, 1990).

14 Keith, T. A. AIMAll (Version 19.10.12). *TK Gristmill Software* (2019).

15 Kazhdan, D. *Coupling of the 4f Electrons in Lanthanide Molecules* Doctor of Philosophy thesis, University of California, Berkeley, (2008).

16 Mehdoui, T., Berthet, J. C. & Ephritikhine, M. Lanthanide(III)/actinide(III) differentiation in coordination of azine molecules to tris(cyclopentadienyl) complexes of cerium and uranium. *Dalton Trans.* **4**, 579-590 (2004).

17 Deacon, G. B., Gatehouse, B. M., Platts, S. N. & Wilkinson, D. L. Organolanthanoids. XI. The Crystal and Molecular Structures of Two Tris(η^5-cyclopentadienyl)(pyridine)-lanthanoid(III) Compounds. *Aust. J. Chem.* **40**, 907-914 (1987).

18 Formanuik, A. *et al.* Double Reduction of 4,4'−Bipyridine and Reductive Coupling of Pyridine by Two Thorium(III) Single-Electron Transfers. *Chem. Eur. J.* **23**, 2290-2293 (2017).
